# Investigating phenotypic plasticity due to toxicants with exposure disparities in primary human breast cells *in vitro*

**DOI:** 10.3389/fonc.2024.1411295

**Published:** 2024-06-10

**Authors:** Jade Schroeder, Katelyn M. Polemi, Anagha Tapaswi, Laurie K. Svoboda, Jonathan Z. Sexton, Justin A. Colacino

**Affiliations:** ^1^ Department of Environmental Health Sciences, University of Michigan, Ann Arbor, MI, United States; ^2^ Department of Pharmacology, University of Michigan, Ann Arbor, MI, United States; ^3^ Department of Medicinal Chemistry, University of Michigan, Ann Arbor, MI, United States; ^4^ Department of Internal Medicine, University of Michigan, Ann Arbor, MI, United States; ^5^ Department of Nutritional Sciences, University of Michigan, Ann Arbor, MI, United States; ^6^ Program in the Environment, University of Michigan, Ann Arbor, MI, United States

**Keywords:** breast cancer, triple negative breast cancer, phenotypic placticity, immunocytochemistry, toxicology, environment, disparities

## Abstract

**Introduction:**

Breast cancer is the second most diagnosed cancer, as well as the primary cause of cancer death in women worldwide. Of the different breast cancer subtypes, triple-negative breast cancer (TNBC) is particularly aggressive and is associated with poor prognosis. Black women are two to three times more likely to be diagnosed with TNBCs than white women. Recent experimental evidence suggests that basal-like TNBCs may derive from luminal cells which acquire basal characteristics through phenotypic plasticity, a newly recognized hallmark of cancer. Whether chemical exposures can promote phenotypic plasticity in breast cells is poorly understood.

**Methods:**

To investigate further, we developed a high-content immunocytochemistry assay using normal human breast cells to test whether chemical exposures can impact luminal/basal plasticity by unbiased quantification of keratin 14 (KRT14), a basal-myoepithelial marker; keratin 8 (KRT8), a luminal-epithelial marker; and Hoechst 33342, a DNA marker. Six cell lines established from healthy tissue from donors to the Susan G. Komen Normal Tissue Bank were exposed for 48 hours to three different concentrations (0.1μM, 1μM, and 10μM) of eight ubiquitous chemicals (arsenic, BPA, BPS, cadmium, copper, DDE, lead, and PFNA), with documented exposure disparities in US Black women, in triplicate. Automated fluorescence image quantification was performed using Cell Profiler software, and a random-forest classifier was trained to classify individual cells as KRT8 positive, KRT14 positive, or hybrid (both KRT8 and KRT14 positive) using Cell Profiler Analyst.

**Results and discussion:**

Results demonstrated significant concentration-dependent increases in hybrid populations in response to BPA, BPS, DDE, and PFNA. The increase in hybrid populations expressing both KRT14 and KRT8 is indicative of a phenotypically plastic progenitor-like population in line with known theories of carcinogenesis. Furthermore, BPA, BPS, DDE, and copper produced significant increases in cell proliferation, which could be indicative of a more malignant phenotype. These results further elucidate the relationship between chemical exposure and breast phenotypic plasticity and highlight potential environmental factors that may impact TNBC risk.

## 1 Introduction

Breast cancer alone accounts for 31% of all new cancer diagnoses in women, and incidence rates have been increasing by approximately 0.5% each year (1). Breast cancer is also responsible for 15% of all female cancer-related deaths each year; however, there are stark contrasts in outcomes and survival across races and ethnicities (1). Despite a 4% lower incidence rate compared to non-Hispanic White women, non-Hispanic Black women have a 40% higher breast cancer-associated mortality rate compared to non-Hispanic White women (1). Furthermore, relative to non-Hispanic White women, non-Hispanic Black women have a two to three times higher risk of developing triple-negative breast cancer (TNBC), an extremely aggressive and heterogenous subtype of breast cancer with no targeted therapy (2).

There is no current data at a molecular or biological level that can fully explain the etiology of these disparities (3). Environmental factors, such as chemical exposure disparities, may play a role in the disparate incidence of TNBC in African American women. However, these drivers are poorly understood. Compared to other demographics, African American women, on average, are exposed to elevated levels of multiple toxicants, including lead, cadmium, arsenic, p,p′-dichlorodiphenyldichloroethylene (DDE), bisphenol S (BPS), and perfluorononanoic acid (PFNA) (4–6). Additionally, bisphenol A (BPA) levels are higher in lower-income individuals, and African American women are more likely to face socioeconomic adversity (7, 8). Many of these disparate exposures can be directly linked to unequal living conditions, associated with historical systemically racist practices, such as redlining, that do not provide an adequate amount of safety and protection against environmental exposures (9). Dietze et. al (3) proposed that neighborhood level factors may be the intersection between disparities and the aggressive nature of TNBC in African American women. Independent of socioeconomic factors, unjust beauty norms result in further disparate exposures from harmful chemicals in targeted personal care products (10). Hair texture preference and colorism have led to widescale production of hair straightener and skin lightening beauty products that are often unregulated and filled with deleterious chemicals and contaminants (10). Chemical hair straightening products, in particular, are disproportionately purchased by African American women and have been associated with premature breast development and increased risk of premenopausal breast cancer (10). While the mechanisms implicating disparate chemical exposures as drivers of aggressive breast cancer are poorly understood, it is becoming increasingly apparent that chemical exposures may impact breast cancer risk (11, 12).

A process that contributes to breast cancer progression is phenotypic plasticity, a newly defined Hallmark of Cancer (13, 14). Phenotypic plasticity describes a cell’s ability to transition and acquire another cellular constitution in response to environmental stress (15). The result is a cell that exhibits “hybrid” characteristics, simultaneous expression of phenotypic characteristics of two or more cell types. These hybrid cells can be present in normal mammary tissue; however, they are primarily observed in malignant cells, and have been shown to promote tumorigenesis and metastasis of breast cancer (15, 16). Of TNBC cases, 80.6% are considered basal-like breast cancers (17). Basal-like breast cancers express genes consistent with normal myoepithelial cells such as KRT14 (17). Numerous other genes can be useful as markers for other cell types, such as KRT8, a known luminal marker gene (18). Overlap of KRT14 and KRT8 expression is indicative of hybrid populations (15), and single-cell analyses have revealed that these hybrid “basoluminal” populations increase with age in the normal mammary gland (19). KRT8 and KRT14 are both intermediate filament proteins that not only reflect the epithelial cell type but can also be used to indicate growth and differentiation factors (20). Upregulation of KRT14 has been associated with a more invasive breast cancer phenotype, while increased abundance of KRT8 is common in malignant cells (21, 22).

For estrogen receptor-negative breast cancers, such as TNBC, there is mounting evidence to suggest that chemical exposures may increase risk for breast cancer by inducing phenotypic plasticity. Recent studies have implicated cadmium, arsenic, and BPA as environmental chemicals capable of inducing phenotypic plasticity in normal human breast cells (1–3, 5–7, 9, 11, 12, 14–16, 18, 19, 22–49). The goal of the present study was to further characterize the association between exposure to chemicals with documented exposure disparities and phenotypic plasticity in primary normal breast cells from diverse donors. Based on previous work that highlights known chemical exposure disparities, as well as prior evidence of chemically induced phenotypic plasticity, we hypothesized that chemicals with known exposure disparities in African American women would promote phenotypic plasticity and hybrid states in normal breast epithelial cells. To test this hypothesis, we optimized a novel high-throughput imaging assay using luminal and basal markers in normal human breast cells. This assay was employed to examine phenotypic plasticity in response to chemical dosing in primary breast cells and further elucidate the environmental factors and disparities present in breast cancer.

## 2 Methods

We developed a high-throughput immunocytochemistry assay to quantify KRT8 and KRT14 staining of toxicant-treated and negative control/vehicle-treated primary human cell lines. The toxicants used were lead acetate (Sigma-Aldrich 316512), copper chloride (Sigma-Aldrich 222011), cadmium chloride (Sigma-Aldrich 202908), sodium arsenite (Sigma-Aldrich S4700), p,p′-DDE (Chem Service N-10875), BPA (Sigma-Aldrich 239658), BPS (Sigma-Aldrich 103039), and PFNA (Sigma-Aldrich 394459). Chemicals were chosen based on a previous study that identified known exposure disparities between non-Hispanic Black women and non-Hispanic White women (5). A diverse set of normal human breast cell lines grown from normal breast punch biopsy tissues obtained from the Susan G. Komen Tissue Bank were used to test these exposures and observe differences in response to chemical dosing *in vitro*. The samples were all from nulliparous women who either self-identified as African American or European American (three cell lines for each group) and matched for age, BMI, and date of last menstrual period (**Supplementary Table 1**).

Following optimization of our novel assay, we cultured each cell line and treated cells with human-relevant concentrations of our chosen chemicals. Following an incubation period, we employed our immunostaining protocol and imaged plates on a high-content imaging microscope. To quantify and analyze dosing effects, as well as interindividual differences, we created Cell Profiler Analyst pipelines. Quantitative analysis and data visualization were completed in R Studio software using the results obtained from Cell Profiler.

### 2.1 Cell culture

Cell lines (**Supplementary Table 1**) were established from tissue by enzymatic and mechanical digestion as previously described (15, 50). The resulting cryopreserved cell lines were thawed and cultured in accordance with previously established methods (15, 38). Primary cells grew to confluence in T-75 flasks with irradiated mouse J2 fibroblasts, which provide an optimal growth environment (15).

Once confluent, cells were diluted to 50,000 live cells per ml in F-media, to be plated at 1,500 cells (30 μl) per well in collagen-coated 384-well plates (Corning Biocoat Collagen I-rat tail collagen type I Product Number: 354667). Cells were plated 24 h prior to dosing and placed in a humidified incubator at 37°C/5% CO2 overnight.

F-media was prepared by combining 500 ml of DMEM (Fisher, cat. no. 11965092), 50 ml of heat-inactivated fetal bovine serum (Sigma Aldrich, cat. no. F4135), 5.5 ml of 200 mM L-glutamine (Gibco, cat. no. 25–030-081), 5.5 ml of 100X Pen-Strep (Fisher, cat. no. 15140122), 187 ml of F-12 (Fisher, cat. no. 11765054), 194.48 μl of 96 μg/ml of hydrocortisone (Stem Cell, cat. no. 07925), 935 μl of 4 mg/ml of insulin (Fisher, cat. no. 12585014), 8.98 μl of 10 μg/ml of EGF (Stem Cell, cat. no. 78006.1), 62.83 μl of 1.2 μM cholera toxin (Sigma Aldrich, cat. no. C8052), and 623.83 μl of 12 mM Y-27632 inhibitor (Stem Cell cat. no. 72302).

### 2.2 Chemical dosing

Chemical concentrations were prepared using serial dilutions, and each chemical was diluted to three concentrations: 100 nM, 1 μM, and 10 μM. These concentrations were chosen given established biological relevance from previous work from our group that established benchmark concentrations *in vitro* linked to biomarker concentrations from human population data from the National Health and Nutrition Examination Survey (6, 22). Sala-Hamrick et al. established median benchmark concentrations for each of the toxicants used in this study using RNA sequencing and found large impacts between 10 nM and 10 μM (22). Water (Invitrogen 10–977-015) and DMSO (Sigma-Aldrich D2650) served as the vehicles for heavy metals and organics, respectively. Three replicates were assessed per concentration for each chemical on each cell line. Following dosing, well plates were placed back in the humidified incubator at 37°C/5% CO_2_ for 48 h prior to immunostaining.

### 2.3 Immunostaining and imaging

After exposure, cells were stained for expression of KRT8 and KRT14, along with the nuclear stain Hoechst 33342. The following reagent concentrations have been optimized and were used for each cell line: 4% paraformaldehyde was prepared by diluting 925 μl of 16% paraformaldehyde (Thermo Fisher Scientific cat. no. AA433689M) in 3,150 μl of PBS (Gibco cat. no. 10–010-049). PBST was prepared by adding 50 μl of 100% Tween20 (Thermo Fisher Scientific cat. no. BP337) to 49.95 ml of PBS. Triton X (0.1%) was prepared by diluting 3.7 μl of 100% Triton X (Sigma-Aldrich cat. no. T8787) in 3.7 ml of PBST. Blocking buffer was prepared by dissolving 83.33 mg of glycine (Thermo Fisher Scientific cat. no. AAA1381636) in 3.7 ml of PBST and 493.3 μl of 7.5% BSA (Gibco cat. no. 50–121-5315). BSA (1%) was prepared by adding 493.3 μl of BSA to 3.7 ml of PBST. The antibody solution was prepared by adding 24.6 μl of Anti-Cytokeratin 8 (1:150 ratio) (Alexa Fluor 488, Clone number EP 1628Y; Isotype IgG) and 37 μl of Anti-Cytokeratin 14 (1:100 ratio) (Alexa Fluor 647, Clone number EP 1612Y; Isotype IgG) to 3.7 ml of 1% BSA in PBST. The counterstaining solution was prepared by adding 1.9 μl of Hoechst 33342 (Thermo Fisher Scientific cat. no. H3570) and 3.7 ml of 1% BSA in PBST.

After 48 h of incubation (37°C), dosed cells were washed with PBS and then fixed with 4% paraformaldehyde in PBS (pH 7.4). 40 μl of 4% paraformaldehyde was dispensed into each well, and the plate was centrifuged once at 500*g* for 30 s. Following centrifugation, plates were allowed to sit at room temperature for 10 min. The 4% paraformaldehyde was then aspirated, and cells were washed with PBS again prior to permeabilization. Cells were permeabilized with 0.1% Triton-X 100 in PBS. Triton-X 100 (0.1%) was also dispensed at 40 μl per well, and the plate was centrifuged again at 500*g* for 30 s. The plate then sat at room temperature for 10 min before being washed twice with PBS. To prevent any non-specific staining, cells were blocked using a 1% BSA and glycine in PBST blocking buffer. 40 μl of blocking buffer was added to each well, the plate was centrifuged at 500*g* for 30 s, and then sat at room temperature for 1 h. The cells were not washed after blocking; the buffer was aspirated, and 40 μl per well of the antibody solution was added. The plate was centrifuged once more at 500*g* for 30 s and then covered and allowed to incubate overnight (8–12 h) in a 4°C refrigerator. The next day, the antibody solution was decanted, and cells were washed with PBST three times in the dark prior to counterstaining. 40 μl of the counterstaining solution was added to each well, and the plate was centrifuged at 500*g* for 30 s. The plate was then covered and allowed to rest at room temperature for 1 h in the dark. Cells were then washed three times with PBST before imaging with the Yokogawa Cell Voyager 8000 (CV8000) microscope. Automated plate imaging was performed on the CV8000 with a ×20/1.0NA water immersion objective lens, 50-µm pinhole, with three channels; 405 nm (Hoescht), 488 nm (KRT8), and 647 nm (KRT14). Laser power for each channel was adjusted to ensure optimal signal-to-noise ratios, laser-based autofocus was performed at each field, and nine fields per well were imaged.

### 2.4 Fluorescence microscopy analysis

Images obtained from the CV8000 were analyzed using Cell Profiler and Cell Profiler Analyst software (45, 46). To analyze the acquired images, we optimized a quality control (QC) pipeline in Cell Profiler, as well as an analysis pipeline in Cell Profiler. Images ran through the QC pipeline first, and a gentle boosting classifier was created to identify images that were oversaturated for each cell line in Cell Profiler Analyst to create unique flag rules. These rules were then inputted into the Cell Profiler Analysis pipeline, and oversaturated images were removed and excluded from further analysis. In the Cell Profiler Analysis pipeline, nuclei were set as primary objects, while an overlay of both keratins was used to identify individual cells as secondary objects. Mean intensity of KRT8 and KRT14 were measured on a per-cell basis to ensure population classification was accurate in the Cell Profiler classification. Total cell counts were quantified using the primary object, including any cells that did not express either keratin protein.

Following the completion of the analysis pipeline, we trained a random forest classifier for each cell line in Cell Profiler Analyst to recognize and identify the different antibody staining patterns, as well as any remaining J2 fibroblasts, which were identified by Hoechst and their unique nuclear staining. Classifiers were trained to at least 90% accuracy. These classifiers were specifically grouped into four categories: luminal (KRT8+/KRT14−), myoepithelial (KRT14+/KRT8−), hybrids (KRT8+/KRT14+), and cells that did not express either protein (KRT8−/KRT14−) (**Supplementary Figure 1**).

Output from classifications were saved as CSV documents that scored each image and identified the number of each phenotype across all treatments and cell lines. This CSV text document was then inputted into R software (1.4.1717) for further analysis and data visualization.

### 2.5 Data analysis

**Figure 1:**
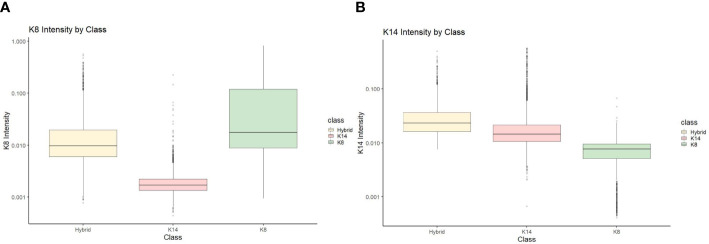
KCR 8195 classification intensity validation for **(A)** KRT8 intensity and **(B)** KRT14 intensity.

To perform statistical analysis, CSV documents generated from Cell Profiler Analyst were uploaded and used to compare the proportion of cells that fluoresced in each category (KRT8, KRT14, and hybrids) by treatment and concentration. J2 fibroblasts were excluded from further analysis, although primary cells that were negative for either KRT8 or KRT14 were still accounted for in the total cell counts. To ensure the accuracy of the Cell Profiler Analyst classifier, validation of the classified cells (KRT8, KRT14, and hybrid) were verified by comparing KRT8 and KRT14 staining intensity data (**Figure 1** shows an example of this for one line, KCR8195. **Supplementary Figures 2–6** show the validation data from the remaining five lines.).

Wilcoxon signed rank-sum tests were utilized to quantify differences in the proportions of KRT8, KRT14, and hybrids by treatment and concentration for a given cell line, compared to the appropriate vehicle control. Significance was measured using Wilcoxon signed rank-sum tests (p < 0.05), and three criteria were used to indicate phenotypic plasticity: 1. Significant increases in hybrid populations, and/or 2. Significant and complementary shifts between hybrid populations and KRT8 or KRT14 populations, and/or 3. Significant and complementary shifts between KRT8 and KRT14 populations. Conditions that met at least one of the three criteria were indicators of phenotypic plasticity in this study. Cell count was also compared by treatment and concentration by conducting Wilcoxon signed rank-sum tests. Significant decreases in hybrid populations without a concomitant increase in another cell population were not counted as phenotypic plasticity; while some phenotypic plasticity may be present, changes in total cell count after dosing could have also contributed to population decreases.

## 3 Results

### 3.1 Concentration-response effect on cell populations with fluorescence microscopy

**Figure 2:**
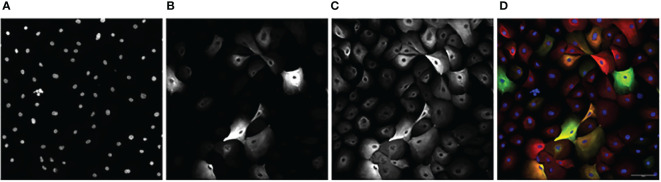
An example image series of KCR 8195 dosed with 1 μM of PFNA: **(A)** Nuclei. **(B)** KRT8. **(C)** KRT14 **(D)** Composite with KRT14 immunofluorescence shown in red, KRT8 immunofluorescence shown in green, with Hoechst shown in blue. Orange and yellow cells represent KRT8/KRT14 basoluminal hybrids. Scale bar represents 100 μm.

Images obtained from the CV8000 were analyzed for nuclear (Hoescht), luminal (KRT8), myoepithelial (KRT14), and hybrid (KRT8/KRT14 overlap) expression (**Figure 2**).

**Figure 3:**
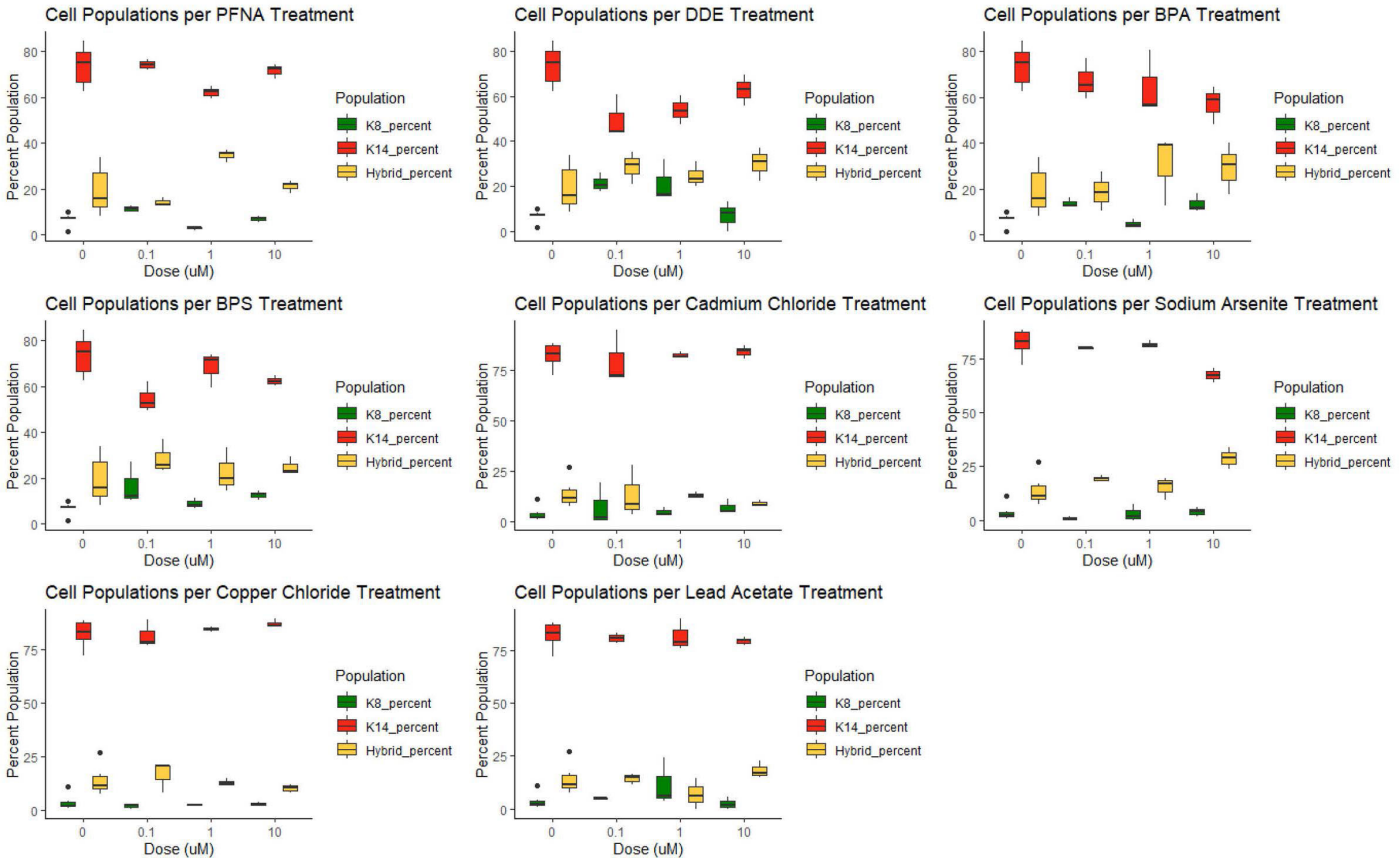
KCR 8195 combined populations (%) for each chemical compared to each associated control (0 μM concentration). Significance determined by Wilcoxon signed rank-sum tests and reported in **Figures 4**, **5** and **Supplementary Figures 12–15**.

KCR7889, KCR7953, KCR8195, and KCR8580 primarily expressed myoepithelial cells in both the dosed and control populations (**Figure 3** and **Supplementary Figures 8, 9** and **Supplementary Figure 11**). KCR7518 and KCR8519 primarily expressed luminal cells in both the dosed and control populations (**Supplementary Figures 7, 10**). Basal populations of hybrid cells were present in each cell line as well. To identify the effect of chemical concentrations on phenotypic plasticity, we used machine learning to quantify the amount of each cell type under each dosed condition and compared these values to controls (**Figure 3** and **Supplementary Figures 7–11**). KCR 8195, in particular, had a high cell count as well as a large presence of hybrid cells at baseline and was subsequently chosen for presentation. As shown in **Figure 3**, KCR 8195 had concentration-dependent decreases in KRT14-marked cells and subsequent increases in KRT8 and hybrid cell populations, with significant shifts seen in low concentrations of p,p′-DDE and BPS.

### 3.2 Significant changes in cell type as a marker for phenotypic plasticity

**Figure 4:**
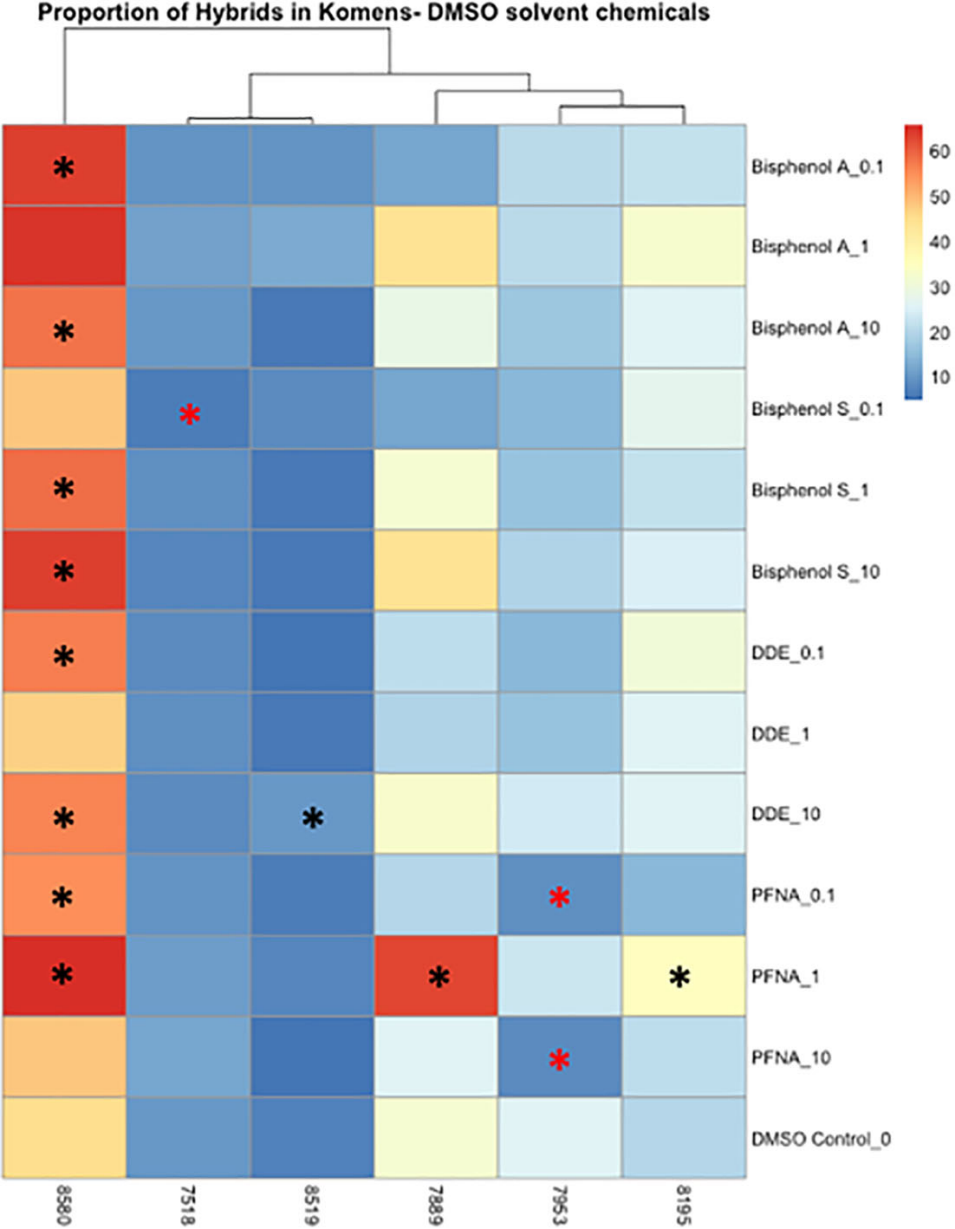
Heatmap depicting the percent of the cells in each treatment condition, which are in a hybrid state, for organic chemical-treated cells only. Differences in hybrid percentages between a given treatment and the DMSO control were determined by Wilcoxon signed rank-sum tests and denoted by an * (p < 0.05). Increases in hybrid populations are represented by a black asterisk, while decreases in hybrid populations are represented by red asterisks.

**Figure 5:**
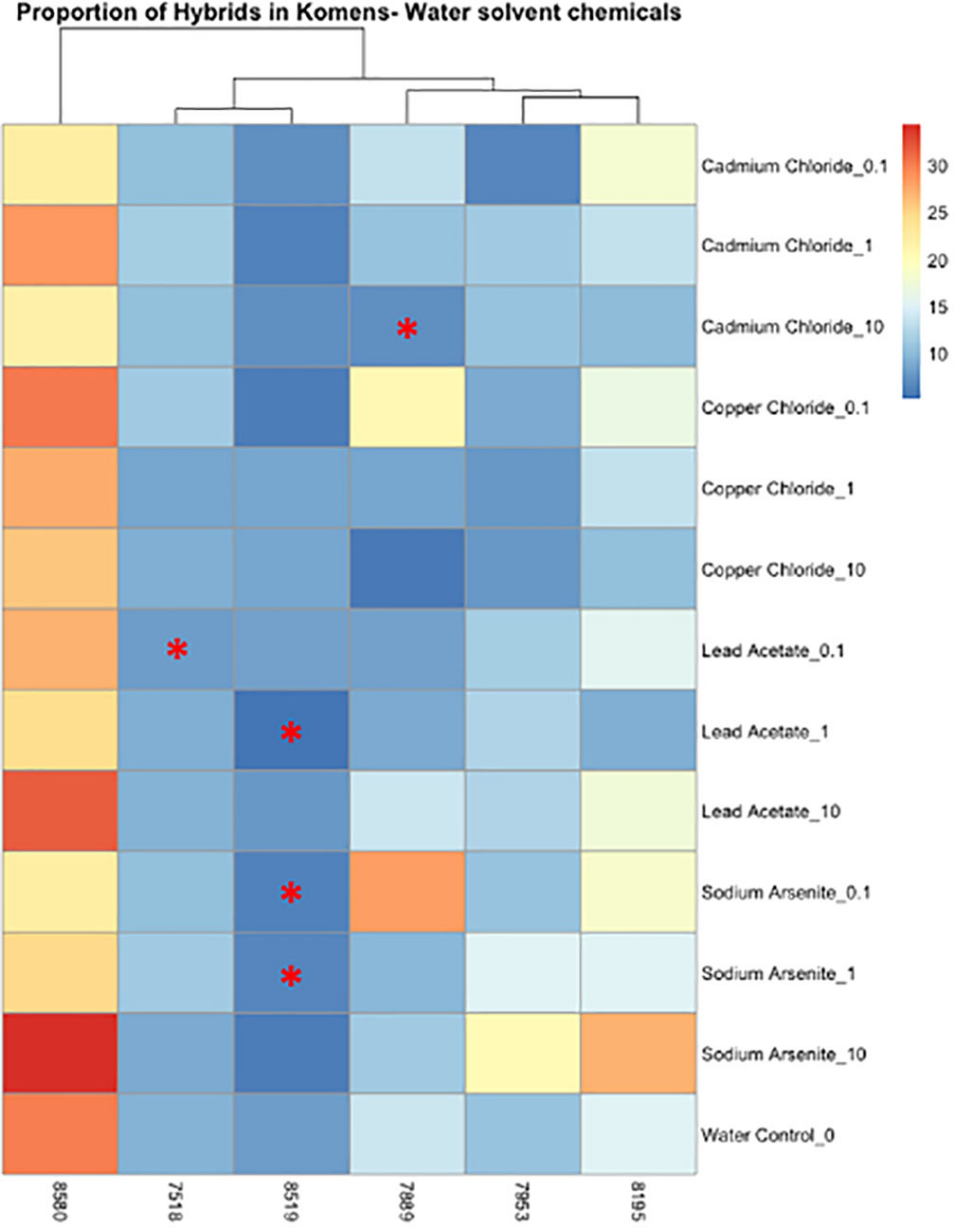
Heatmap depicting the percent of the cells in each treatment condition, which are in a hybrid state, for metal-treated cells only. Differences in hybrid percentages between a given treatment and the water control were determined by Wilcoxon signed rank-sum tests and denoted by a * (p < 0.05). Increases in hybrid populations are represented by a black asterisk, while decreases in hybrid populations are represented by red asterisks.

Significant changes in cell type, either from luminal to myoepithelial, myoepithelial to luminal, or increases and decreases of hybrid populations were used as markers of phenotypic plasticity. Significance was determined using Wilcoxon signed rank-sum tests (p < 0.05). In controls, four of the six cell lines were predominately myoepithelial; however, each displayed significant changes in the KRT8 luminal marker under dosed conditions (**Supplementary Figures 12, 13**). KCR 7889 demonstrated the most myoepithelial to luminal plasticity, with significance found in 10 different concentrations across each of the organic chemicals that were used (**Supplementary Figure 12**). KRT 8580 demonstrated the most phenotypic plasticity, with significant myoepithelial to hybrid shifts as well as luminal to hybrid shifts in eight different concentrations across each of the organic chemicals that were used (**Supplementary Figures 12, 13**). The two cell lines that presented predominately luminal markers basally also demonstrated significant plasticity. Notably, KCR 7518 had a significant shift between hybrid to KRT8 luminal populations in one concentration of BPS, and KCR 8519 had a significant increase in KRT14 myoepithelial cells following exposure to two concentrations of lead acetate (**Supplementary Figures 12, 15**). Overall, most cell lines demonstrated the potential for phenotypic plasticity; however, KCR 8580 had the highest proportion of hybrid cells at baseline and demonstrated the most significant transitions between luminal and myoepithelial cell types (**Figures 4**, **5** and **Supplementary Figures 12–15**).

### 3.3 Changes in cell count as an implication for requisite future work

**Figure 6:**
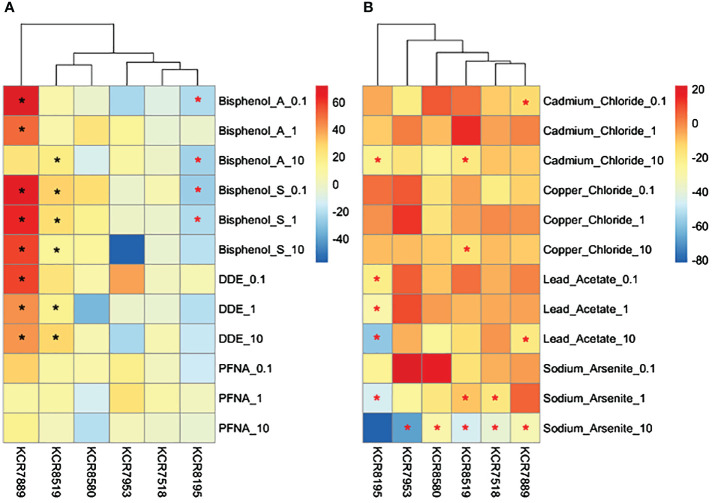
Heatmaps depicting changes in cell counts (% relative to control) per sample for **(A)** DMSO solvent chemicals and **(B)** water solvent chemicals. Chemical concentrations are measured in μM—0.1 is equal to 100 nM. Differences in cell counts relative to control were determined by Wilcoxon signed rank-sum tests and denoted by a * (p < 0.05). Increases in cell counts are represented by a black asterisk, while decreases are represented by red asterisks.

The average cell counts of each cell line varied; however, water control wells and wells that were dosed with metals diluted in water were more robust to cell number changes than DMSO control wells or wells that were dosed with chemicals diluted in DMSO across cell lines (**Figure 6**). Significant decreases in cell number occurred in multiple chemicals at multiple concentrations. KCR 8195 had significant decreases in cell number in 100 nm and 10 μm of BPA, 100 nm and 1 μm of BPS, 1 μm of cadmium, 1 μm of arsenic, and across all lead concentrations (**Supplementary Figure 16**). KCR 7518 had significant decreases in cell number throughout all concentrations of arsenic (**Supplementary Figure 17**). KCR 7889 had significant decreases in cell number in 100 nm of cadmium, 10 μm of arsenic, and 10 μm of lead (**Supplementary Figure 18**). KCR 7953 only saw a significant decrease in cell number in 10 μm of arsenic (**Supplementary Figure 19**). KCR 8519 had significant decreases in cell number in 10 μm of cadmium, 1 and 10 μm of arsenic, and 10 μm of copper (**Supplementary Figure 20**). KCR 8580 only saw a significant decrease in cell number in 10 μm of arsenic (**Supplementary Figure 21**).

## 4 Discussion

There are consistent data that implicates chemical exposures as drivers of breast cancer. Cadmium, arsenic, and BPA have each been associated with epigenetic modifications in normal human breast cells; previous work has demonstrated that each of these chemicals may be drivers of malignant phenotypic plasticity in normal human breast cells via basoluminal transitions (23, 24, 49). In addition to these three chemicals, prior research has highlighted that exposure disparities of lead, p,p′-DDE, PFNA, BPS, and copper exist for African American women (6). In this study, we sought to further characterize the potential for disparate chemical exposures to induce malignant phenotypic plasticity *in vitro*.

Phenotypic plasticity was identified by significant increases in hybrid populations, or through significant and complementary changes between cytokeratin 8 and cytokeratin 14 populations, a summary of which is shown in **Table 1**. Collectively, these findings suggest that arsenic, BPA, BPS, DDE, and PFNA are capable of stimulating basoluminal transitions in normal human breast cells. This is consistent with previous findings that identified low *in vivo* and *in vitro* concentrations, between 7 nM and 2 μM of BPA, 1 nM and 3 μM of BPS, 20 nM to 0.75 μM of DDE, and 1 nM to 1.65 μM of PFNA, could induce transcriptomic changes in breast cells (22).

**Table 1:** Summary of phenotypic plasticity for each cell line as indicated by increases in hybrid populations and/or shifts between KRT 8 and KRT 14 populations, as well as significant total cell count increases or decreases by cell line.

Cell Line	Increase in Hybrids	KRT 8/KRT 14 Shift	Increase in Cell Count	Decrease in Cell Count
KCR 7518	None	Decrease in hybrid populations seen with subsequent increase in KRT 8 populations in 100 nM BPS	None	Decrease in cell count in all concentrations of Arsenic
KCR 7889	Increase in hybrid populations in 1 μM PFNA and 100 nM Arsenic	Decrease in KRT 14 populations seen with subsequent increase in KRT 8 populations in 10 μM BPS	Increase in cell count in 1 μM and 10 μM concentrations of BPA, and across all concentrations of DDE and BPS	Decrease in cell count in 100 nM of Cadmium, 10 μM of Arsenic, and 10 μM of Lead
KCR 7953	None	None	None	Decrease in cell count in 10 μM Arsenic
KCR 8195	Increase in hybrid populations in 1 μM PFNA and 100 nM BPS	Decrease in KRT 14 seen with subsequent increases in KRT 8 populations in 100 nM DDE	None	Decrease in cell count in 100 nM and 10 μM BPA, 100 nM and 1 μM BPS, 1 μM Cadmium, 1 μM Arsenic, and across all Lead concentrations
KCR 8519	Increase in hybrid populations in 10 μM DDE	Decrease in KRT 14 populations seen with subsequent increase in KRT 8 populations in 100 nM BPA	Increase in cell count in 1 μM and 10 μM DDE, 10 μM BPA, and across all concentrations of BPS	Decrease in cell count in 10 μM Cadmium, 1 μM and 10 μM Arsenic, and 10 μM Copper
KCR 8580	Increase in hybrid populations in 100 nM and 10 μM PFNA, 100 nM and 10 μM DDE, 1 μM and 10 μM BPS, and 100 nM and 10 μM BPA	None	None	Decrease in cell count in 10 μM Arsenic

Changes in total cell count in response to exposures are also highlighted in **Table 1**. Collectively, these results may demonstrate the cytotoxicity of arsenic, cadmium, and lead at these concentrations, which are consistent with previous findings (23, 24, 26, 43). Sodium arsenite, in particular, has been shown to induce apoptosis in MCF-7 breast cancer cells at concentrations greater than 5 μM (43). The interindividual response to metal exposure, as reflected by cell numbers being impacted in cultures from some individuals but not others, is also worthy of exploration in future studies to understand the factors that promote or prevent toxic effects from a given substance.

Results shown in **Table 1** may also demonstrate the proliferative effects of BPA, BPS, and DDE, which are consistent with previous findings (22, 30, 42). These proliferative effects have been attributed to genetic and epigenetic modifications following exposures in non-tumorigenic MCF-10A breast epithelial cells, with specific upregulation of human epidermal growth factor following BPS exposure, and upregulation of the PCNA gene following DDE exposure (22, 30, 42). We also recently found that low-concentration DDE (25 nM) activates Wnt signaling in MCF-10A cells, as reflected by increased translocation of beta catenin to the nucleus, which may, in part, explain increased cellular proliferation (47). The mechanisms behind the proliferative effects associated with these chemicals in these normal primary lines from diverse donors is worthy of future exploration.

Interindividual differences were present between cell lines at baseline; some lines were predominately luminal, while others were predominately myoepithelial. Among the cell lines used in this study, those that were predominately myoepithelial or contained more hybrid cells at a baseline level demonstrated increased plasticity compared to the cell lines that contained predominately luminal cells in the control. Significant phenotypic plasticity markers were analyzed for each individual, the three European American cell lines never (KCR 7953) or rarely (one for KCR7518 and three for KCR 7889) had significant plasticity, while the African American cell lines more commonly demonstrated plasticity (two for KCR 8519, three for KCR 8195, and eight for KCR 8580). Although the low sample size dictates that no conclusive data can be deduced regarding TNBC disparities, this represents an approximate 3.25-fold increase in phenotypic plasticity markers among the African American cell lines compared to the European American cell lines. Future work is requisite to validate these findings, to test additional diverse cell lines to further elucidate these trends, and to examine the impact of exposures of longer duration, which more accurately model the chronic nature of many of the exposures under investigation. Additional work should also examine the impacts of these toxicants *in vivo* and in well-characterized human tissue samples, as phenotypic plasticity in culture may not reflect what is possible when cells are constrained in a tissue microenvironment. Future research should also consider the larger exposome of toxicants that each individual may be subjected to, as previous work has identified that mixtures of chemicals may increase the aggression of breast cancer cells and promote additional Hallmarks of Cancer, such as invasion and metastasis (51). As we know that people are exposed to complex mixtures of toxicants, reconstructing chemical mixtures at human relevant concentrations, particularly in the context of exposure disparities, and assessing the effects of the mixtures in primary cell lines would be an exciting and important next step.

Additional work is also necessary to further characterize the mechanisms underlying both the cytotoxicity and cellular proliferation associations found in some of these chemicals as well—this work is currently underway. Additional cytotoxicity assays, in particular, may be useful in elucidating the mechanisms behind the alterations in cell number, and the interindividual variation in these alterations, due to increasing concentrations of arsenic, lead, and cadmium. This work also sets the stage for a mechanistic interrogation of how environmental factors can promote cellular plasticity and cell state transitions, for example, by interrogating epigenetic changes (27). Understanding the biological drivers of luminal–basal phenotypic plasticity, and how toxicants can perturb these processes, would provide substantial insights into how chemicals may impact the risk of aggressive breast cancers. Luminal–basal hybrid cells have been identified in normal breast tissue using single-cell profiling (15, 19, 50). Intriguingly, the proportion of these hybrid cells increases with age suggesting aging-associated alterations—perhaps the accrual of a lifetime of exposure to environmental stressors—can promote the emergence or expansion of these cell populations (19). In cancer, experimental studies suggest that basal breast cancers derive from luminal cell populations (33). Studies of estrogen receptor-positive breast cancer MCF7 cells exposed to arsenic long term *in vitro* highlight suppression of epithelial markers, induction of basal markers, and reduced expression of hormone receptors potentially linked to alterations in signaling of stem cell-associated pathways like Hedgehog (24). Research exploring how exposure to additional chemicals and a broader range of concentrations may drive similar effects in primary cells is now requisite. While concentrations chosen for this study were chosen based on previous benchmark concentration modeling based on RNA-seq data, a broader concentration range could be tested in the future, particularly to understand and quantify low concentration effects and potential non-monotonic concentration responses (22). Assaying additional markers may also prove useful in elucidating the role environmental factors may play in the progression of cancer. For example, expression of CD44, CD24, and ALDH1A3 can quantify epithelial and mesenchymal stem cell states in breast cancer and normal breast tissue (37, 50). Associations drawn from these experiments would further solidify the role environmental factors play in TNBC development linked to dysregulated stemness. Further research focused on the specific mechanisms, including epigenetic changes, by which environmental exposures can promote cellular plasticity will provide key insights into how chemical exposures may promote aggressive breast cancers. Overall, these data support that phenotypic plasticity can be modulated by toxicants with disparate exposures *in vitro* in primary human breast cells providing supporting evidence that exposure to these toxicants may be able to perturb this newly defined Hallmark of Cancer.

## Data availability statement

The original contributions presented in the study are included in the article/**Supplementary Material**. Further inquiries can be directed to the corresponding author.

## Ethics statement

Ethical approval was not required for the studies on humans in accordance with the local legislation and institutional requirements because only commercially available established cell lines were used.

## Author contributions

JS: Conceptualization, Data curation, Formal analysis, Investigation, Methodology, Validation, Visualization, Writing – original draft, Writing – review & editing. KP: Formal analysis, Methodology, Validation, Writing – review & editing. AT: Conceptualization, Formal analysis, Visualization, Writing – review & editing. LS: Writing – review & editing. JZS: Formal analysis, Resources, Writing – review & editing. JC: Conceptualization, Formal analysis, Funding acquisition, Methodology, Project administration, Supervision, Writing – original draft, Writing – review & editing.
