# Supplementary material for: Investigating phenotypic plasticity due to toxicants with exposure disparities in primary human breast cells in vitro
Source: Front Oncol. 2024 Jun 10;14:1411295. doi: 10.3389/fonc.2024.1411295 (PMC11194339; doi:10.3389/fonc.2024.1411295)
Supplement: Supplementary file 1 [file DataSheet_1.docx]

**Supplemental Tables & Figures**

*Supplemental Table 1: Susan G. Komen Primary Human Breast Cell Line Demographic Data*

| Cell Line | Race | Age | BMI | Days Since Last Menstrual Period |
| --- | --- | --- | --- | --- |
| KCR8519 | African American | 25 | 21.3 | 9 |
| KCR8195 | African American | 27 | 27.37 | 10 |
| KCR8580 | African American | 25 | 36.32 | 7 |
| KCR7518 | European American | 27 | 30.6 | 19 |
| KCR7889 | European American | 24 | 20.2 | 17 |
| KCR7953 | European American | 24 | 32.3 | 11 |


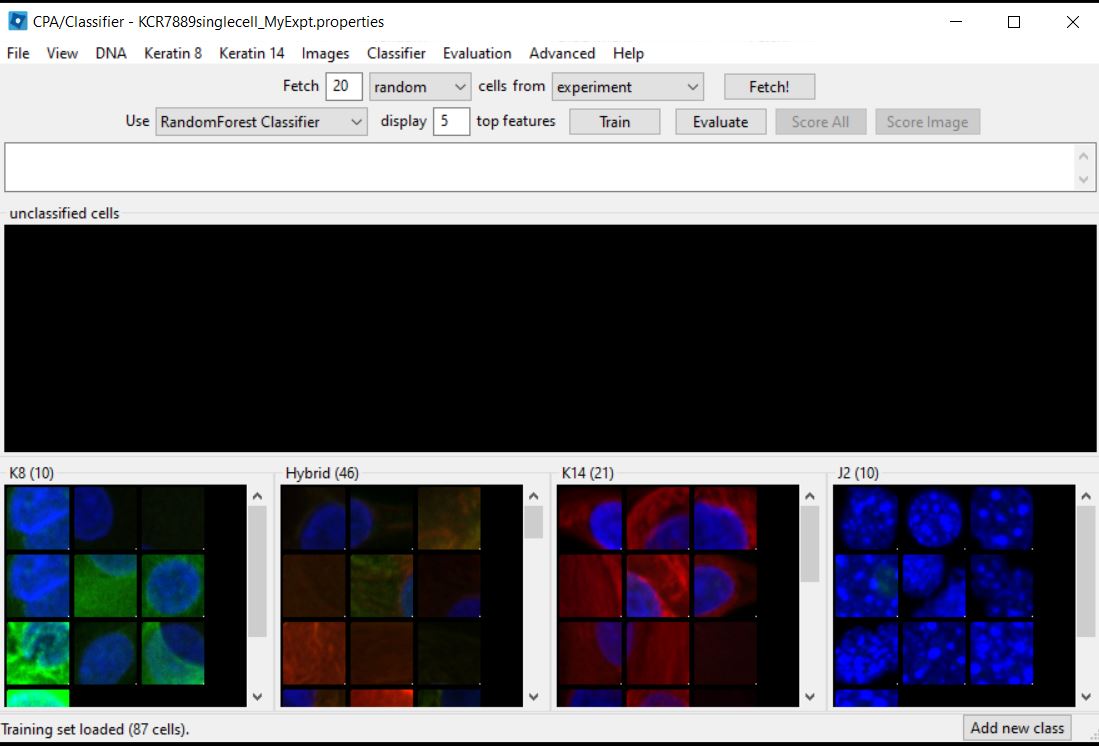


*Supplemental Figure 1: Example of a Cell Profiler Analyst phenotype classifier where single cell images are binned based on their expression for KRT8, KRT14, or if they are hybrids. The J2 bin represents J2 fibroblasts that remained in culture following differential trypsinization.*

*
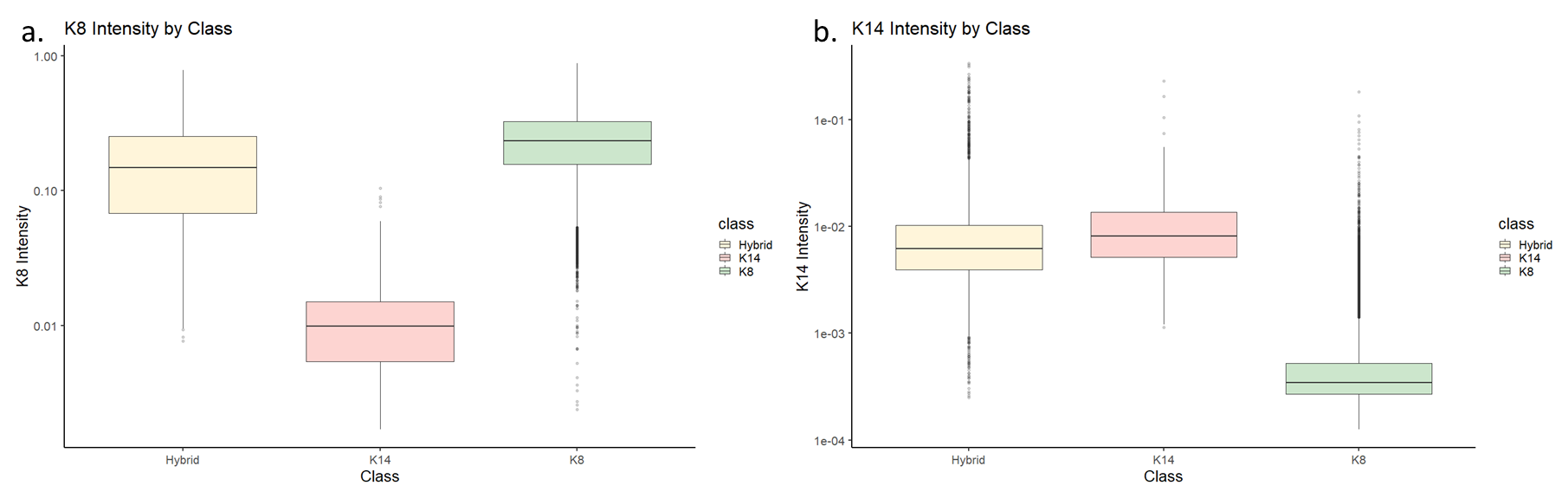
Supplemental Figure 2: KCR 7518 classification intensity validation for a. keratin 8 intensity, and b. keratin 14 intensity.*

*
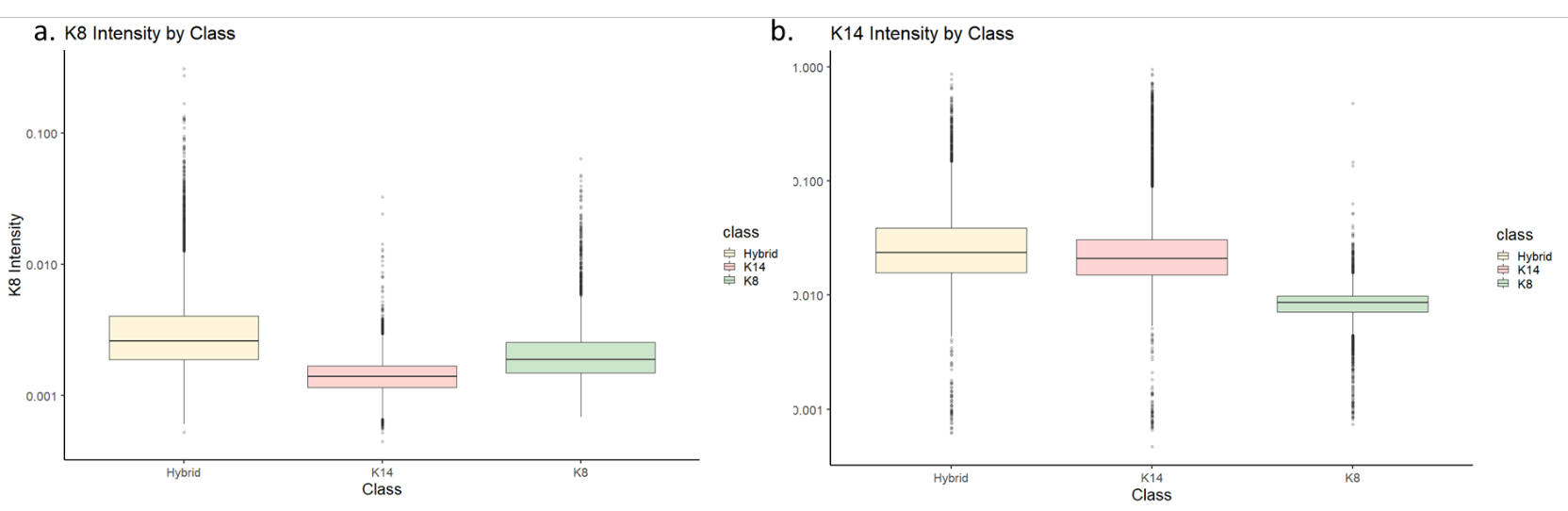
*

*Supplemental Figure 3: KCR 7889 classification intensity validation for a. keratin 8 intensity, and b. keratin 14 intensity.*

*
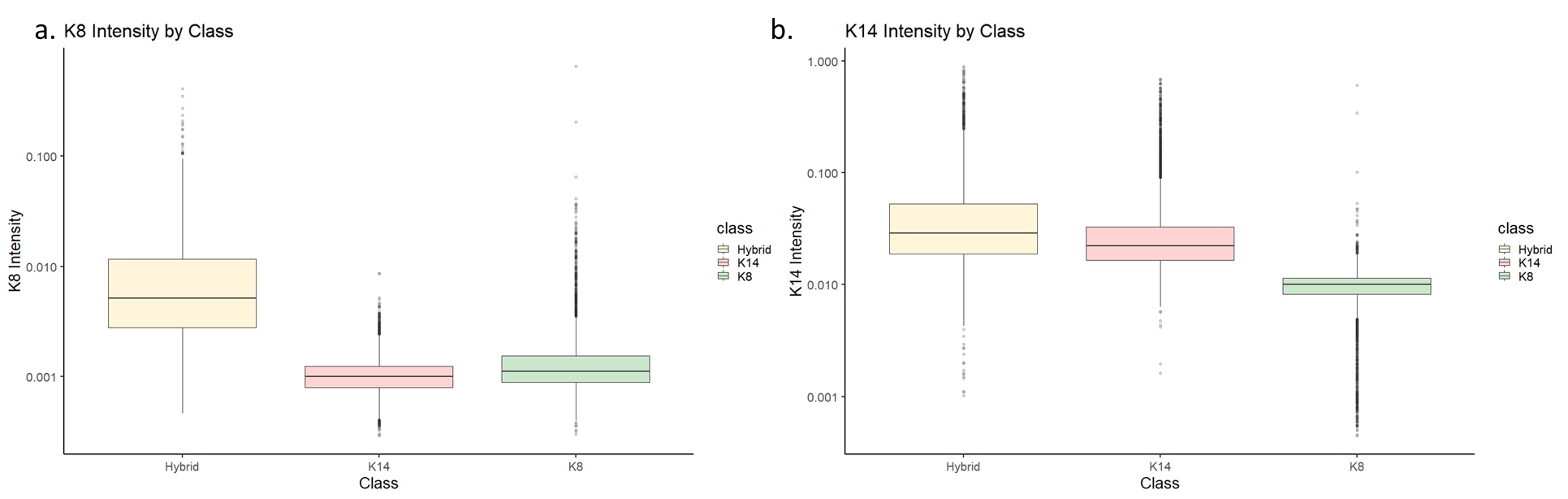
*

*Supplemental Figure 4: KCR 7953 classification intensity validation for a. keratin 8 intensity, and b. keratin 14 intensity.*

*
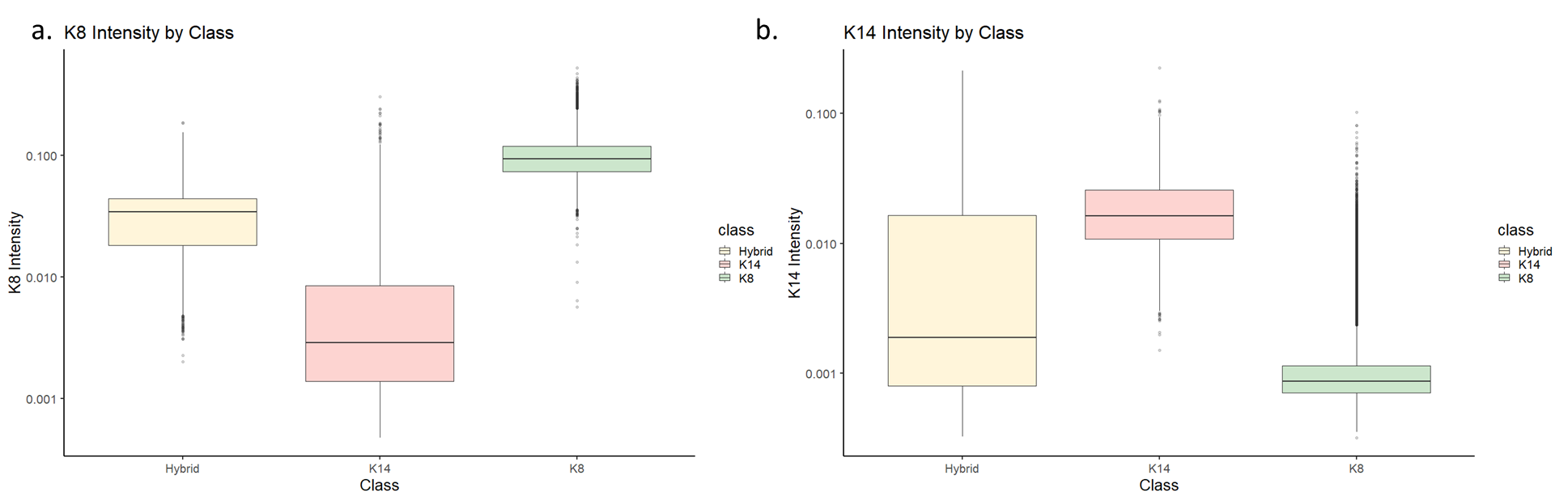
*

*Supplemental Figure 5: KCR 8519 classification intensity validation for a. keratin 8 intensity, and b. keratin 14 intensity.*

*
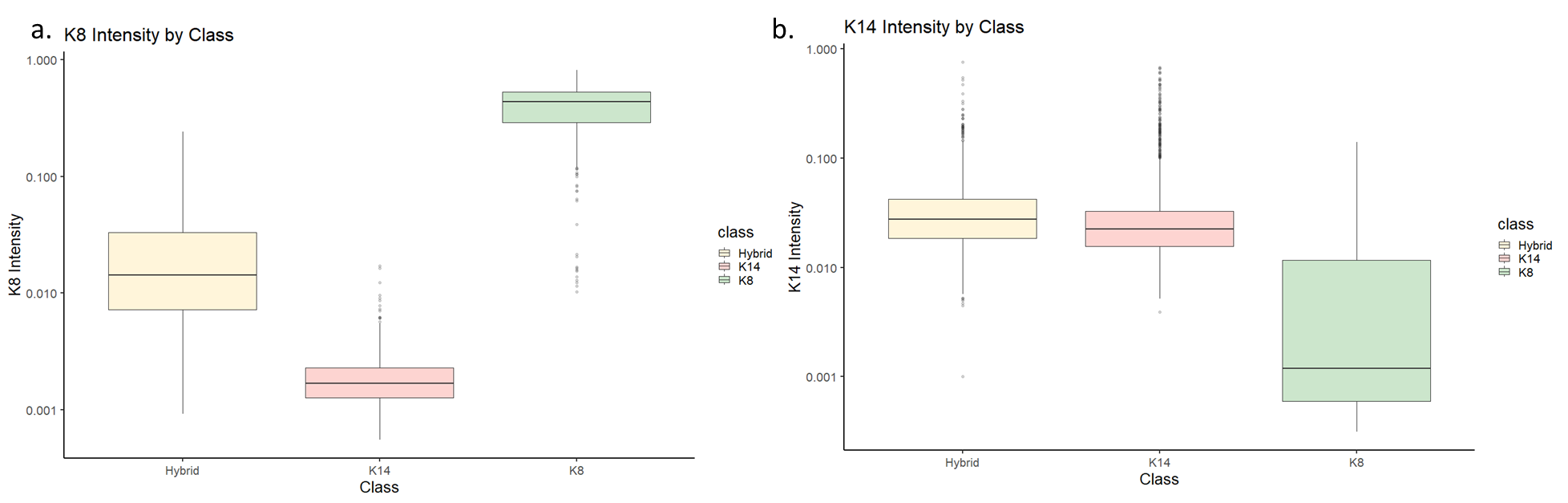
*

*Supplemental Figure 6: KCR 8580 classification intensity validation for a. keratin 8 intensity, and b. keratin 14 intensity.*

*
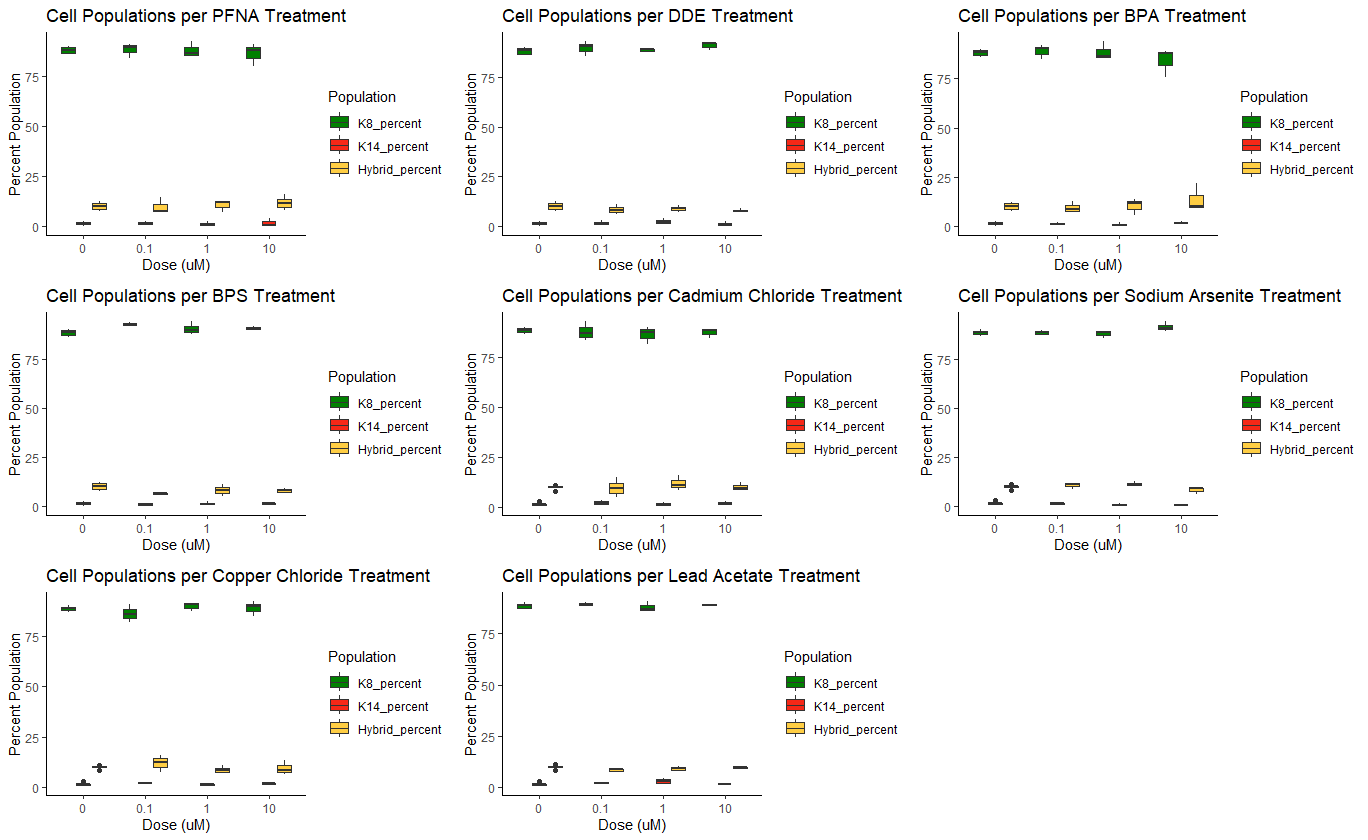
*

*Supplemental Figure 7: KCR 7518 combined populations (%) for each chemical compared to each associated control (0 μM dose). Significance determined by Wilcoxon signed rank-sum tests and reported in Figures 4-5 and Supplemental Figures 12-15.*

**
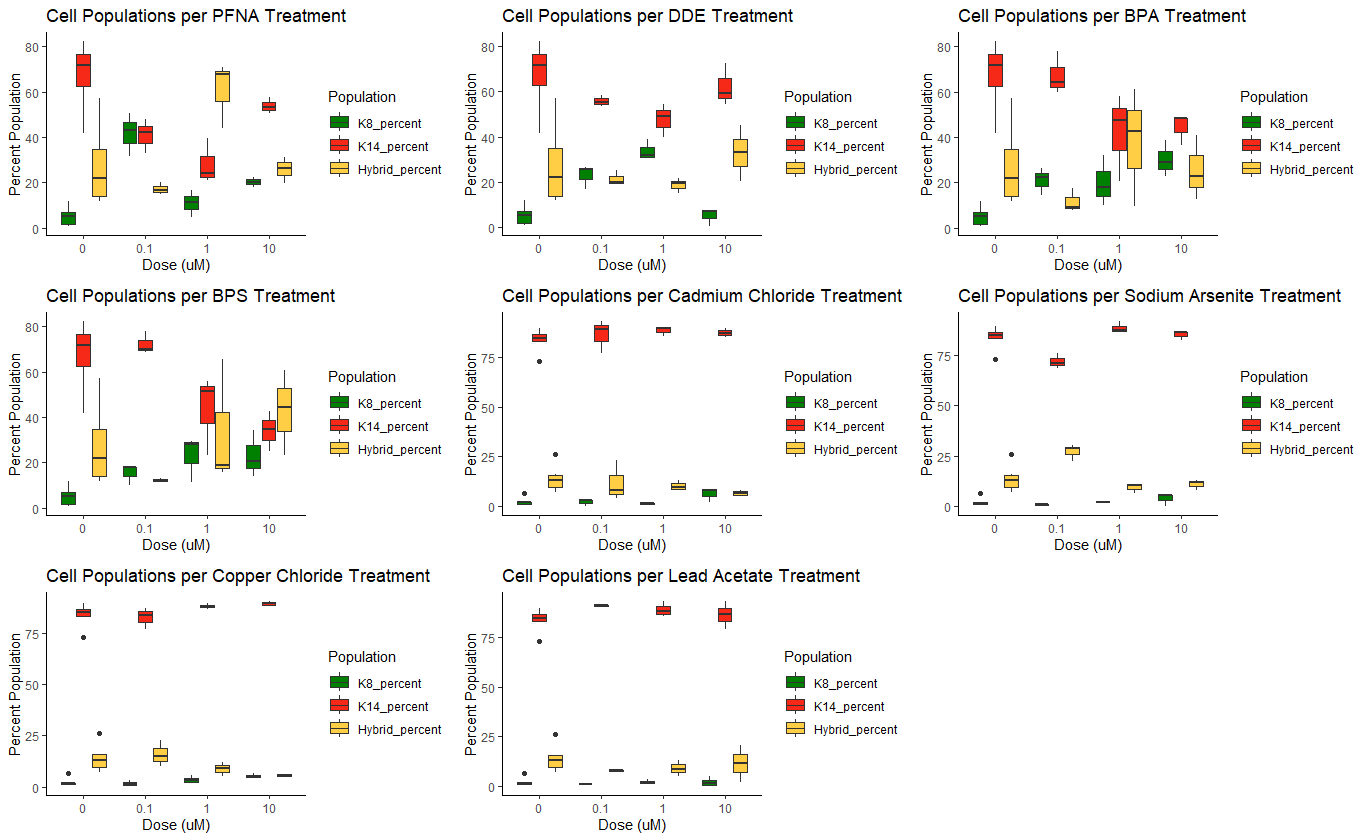
***Supplemental Figure 8: KCR 7889 combined populations (%) for each chemical compared to each associated control (0 μM dose). Significance determined by Wilcoxon signed rank-sum tests and reported in Figures 4-5 and Supplemental Figures 12-15.*

*
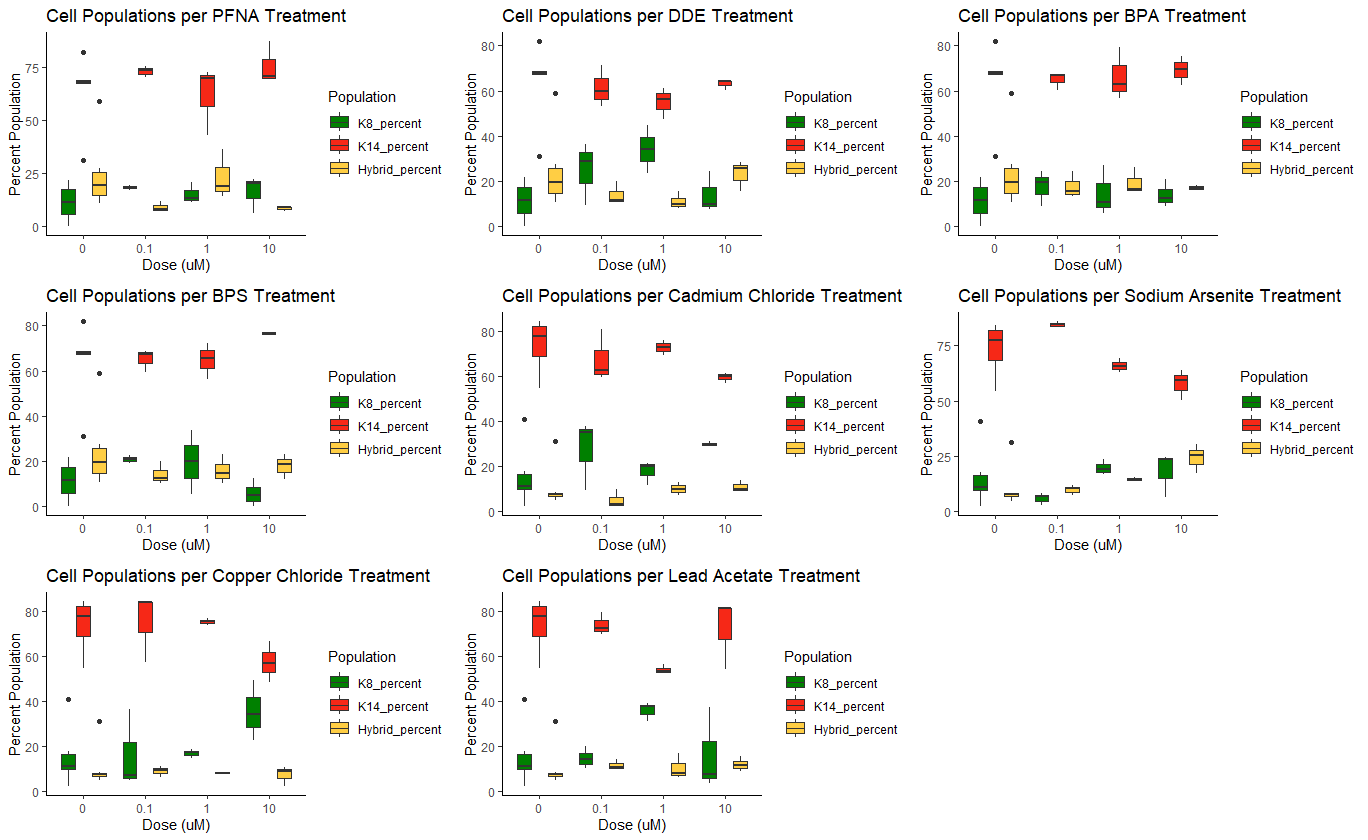

Supplemental Figure 9: KCR 7953 combined populations (%) for each chemical compared to each associated control (0 μM dose). Significance determined by Wilcoxon signed rank-sum tests and reported in Figures 4-5 and Supplemental Figures 12-15.*

**
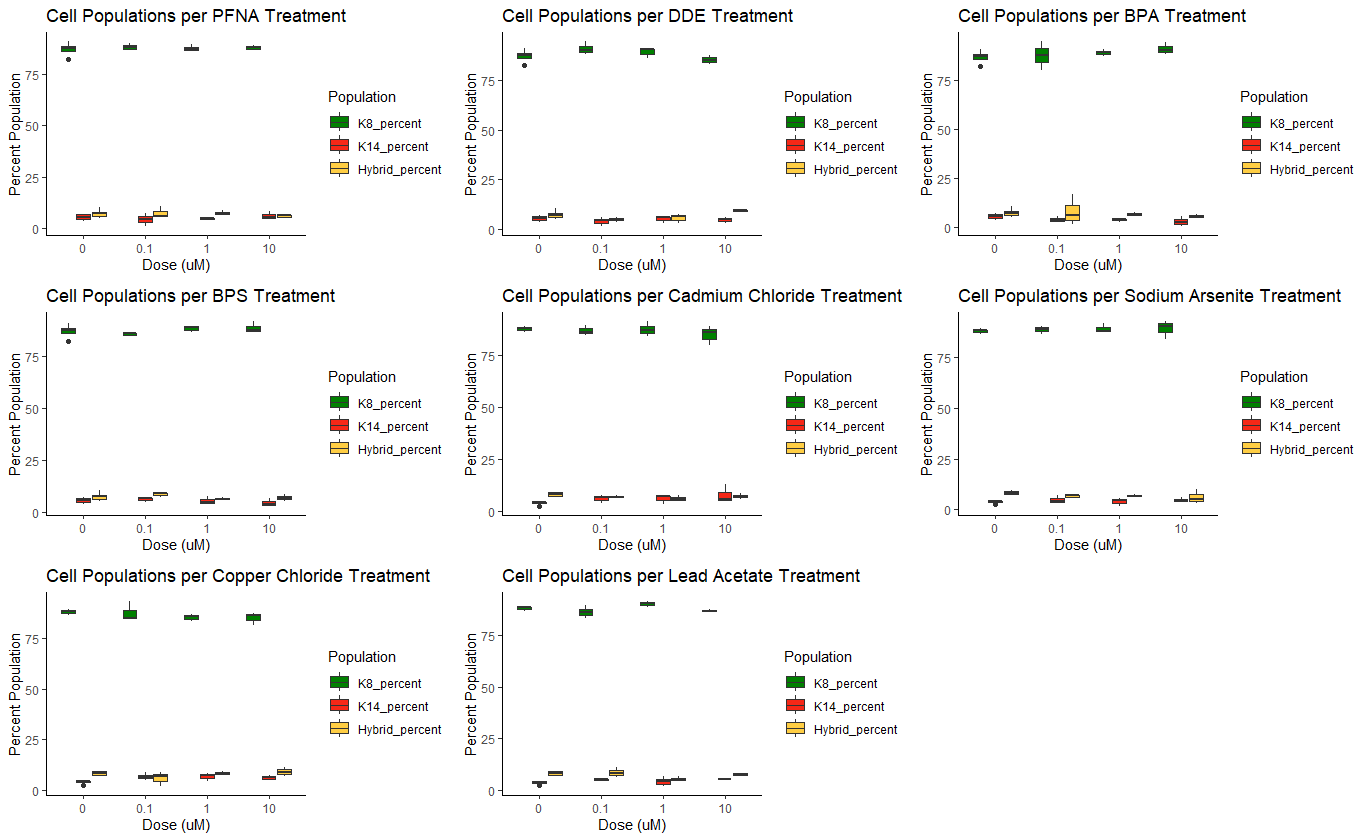
***Supplemental Figure 10: KCR 8519 combined populations (%) for each chemical compared to each associated control (0 μM dose). Significance determined by Wilcoxon signed rank-sum tests and reported in Figures 4-5 and Supplemental Figures 12-15.*


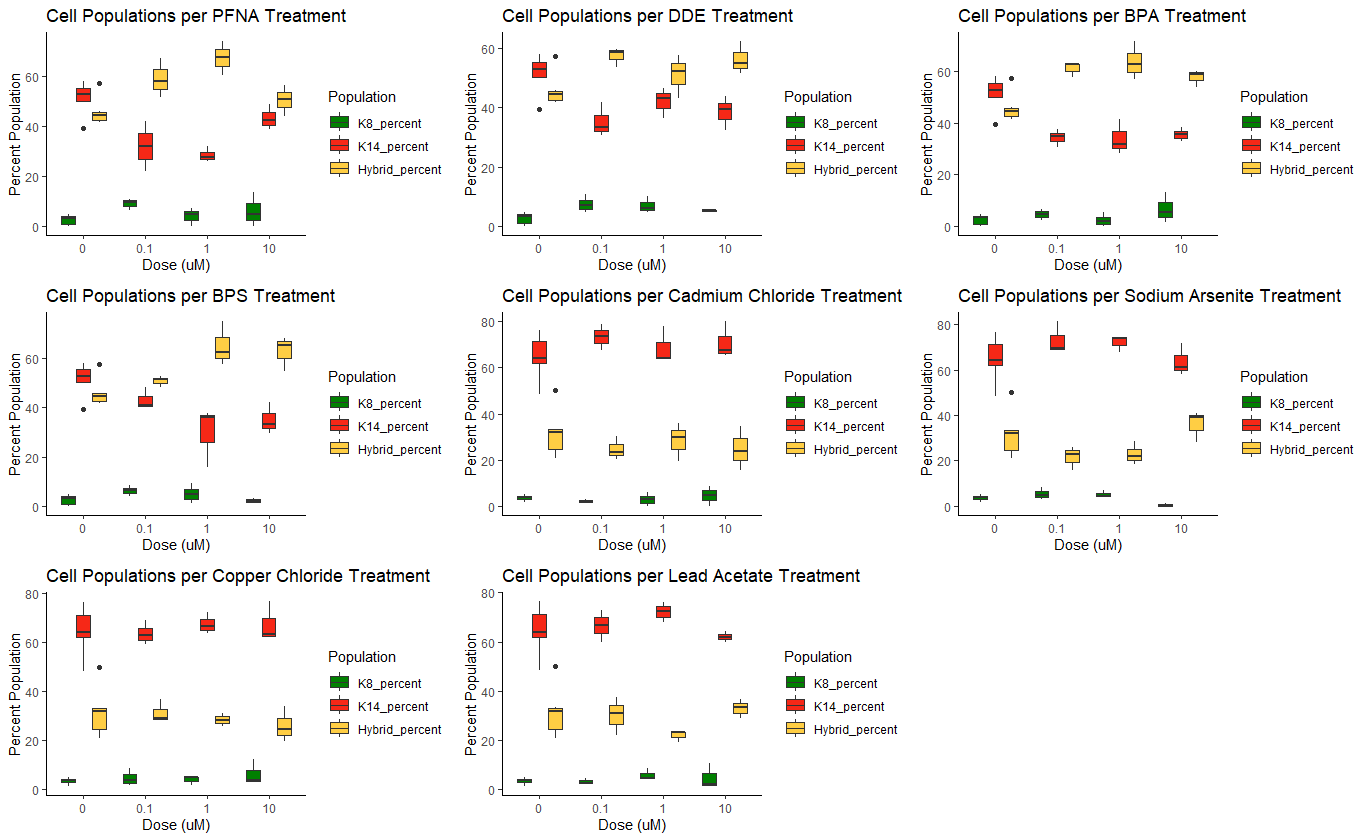


*Supplemental Figure 11: KCR 8580 combined populations (%) for each chemical compared to each associated control (0 μM dose). Significance determined by Wilcoxon signed rank-sum tests and reported in Figures 4-5 and Supplemental Figures 12-15.*

**
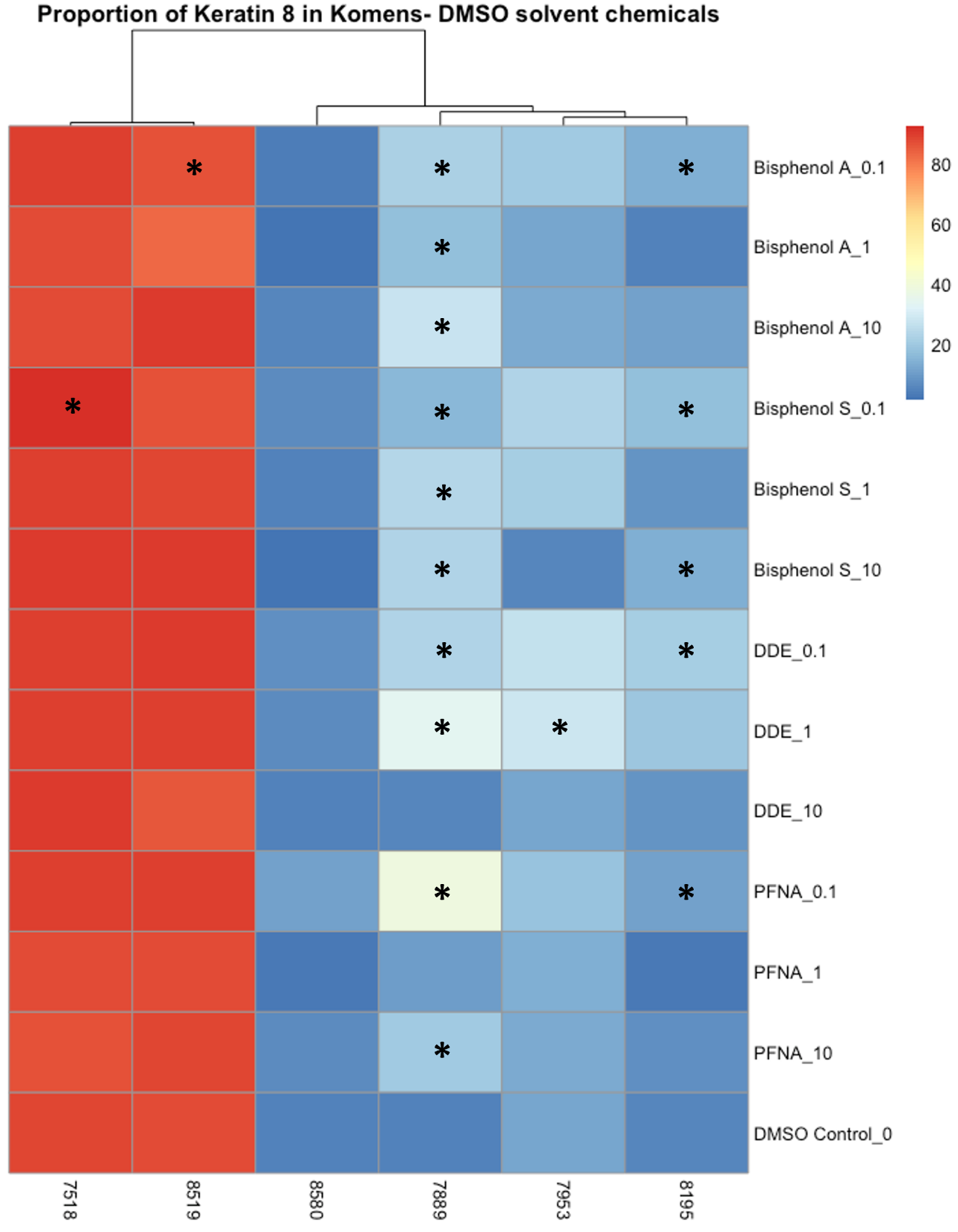
**

*Supplemental Figure 12: Heatmap depicting the percent of the cells in each treatment condition which are in a luminal (KRT8 marked) state, for organic chemical treated cells only. Differences in KRT8 percentages between a given treatment and the DMSO control were determined by Wilcoxon signed rank-sum tests and denoted by a * (p<0.05). Increases in KRT8 populations are represented by a black asterisk, while decreases in KRT8 populations are represented by red asterisks.*

*
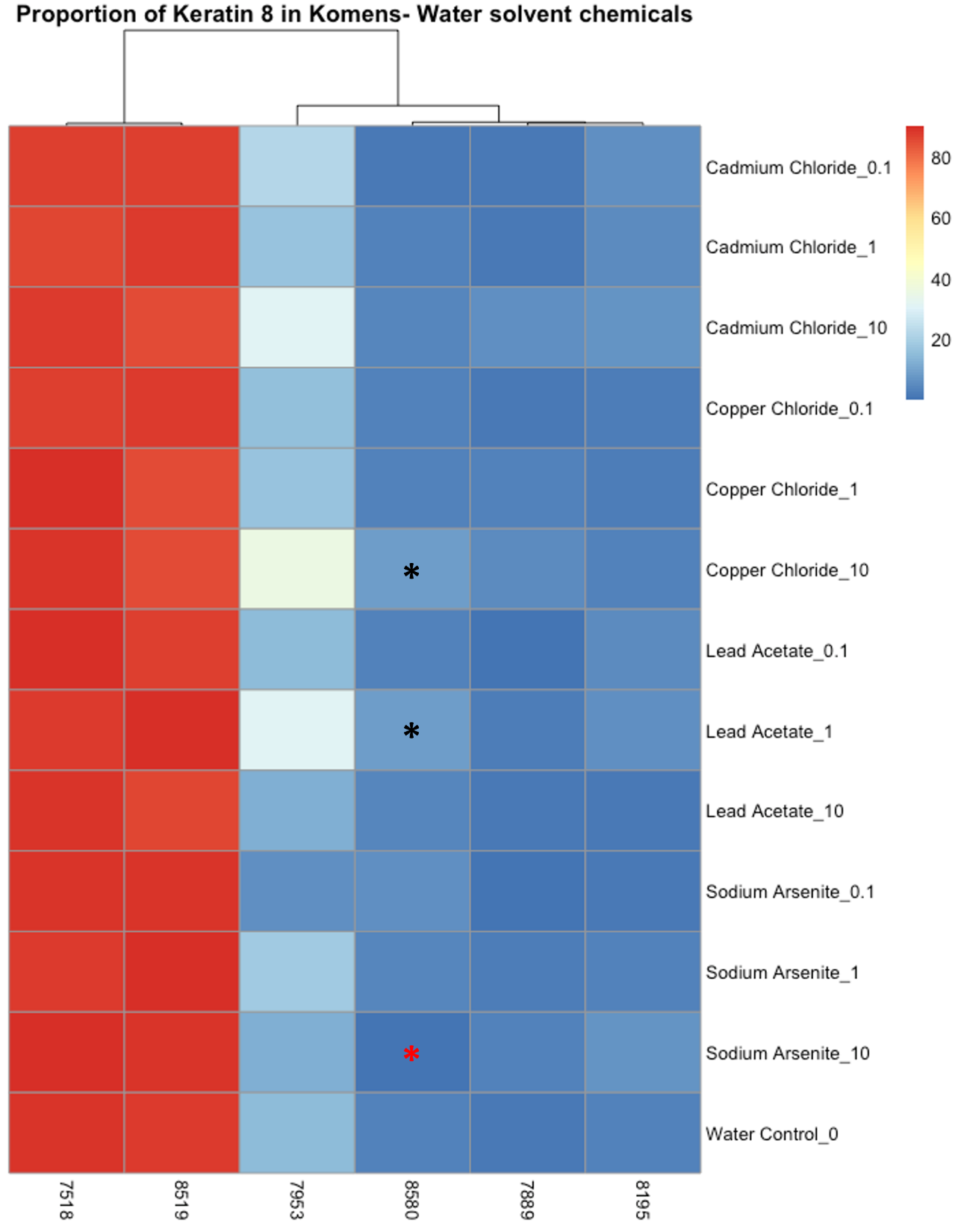

Supplemental Figure 13: Heatmap depicting the percent of the cells in each treatment condition which are in a luminal (KRT8 marked) state, for metal treated cells only. Differences in KRT8 percentages between a given treatment and the water control were determined by Wilcoxon signed rank-sum tests and denoted by a * (p<0.05). Increases in KRT8 populations are represented by a black asterisk, while decreases in KRT8 populations are represented by red asterisks.*

*
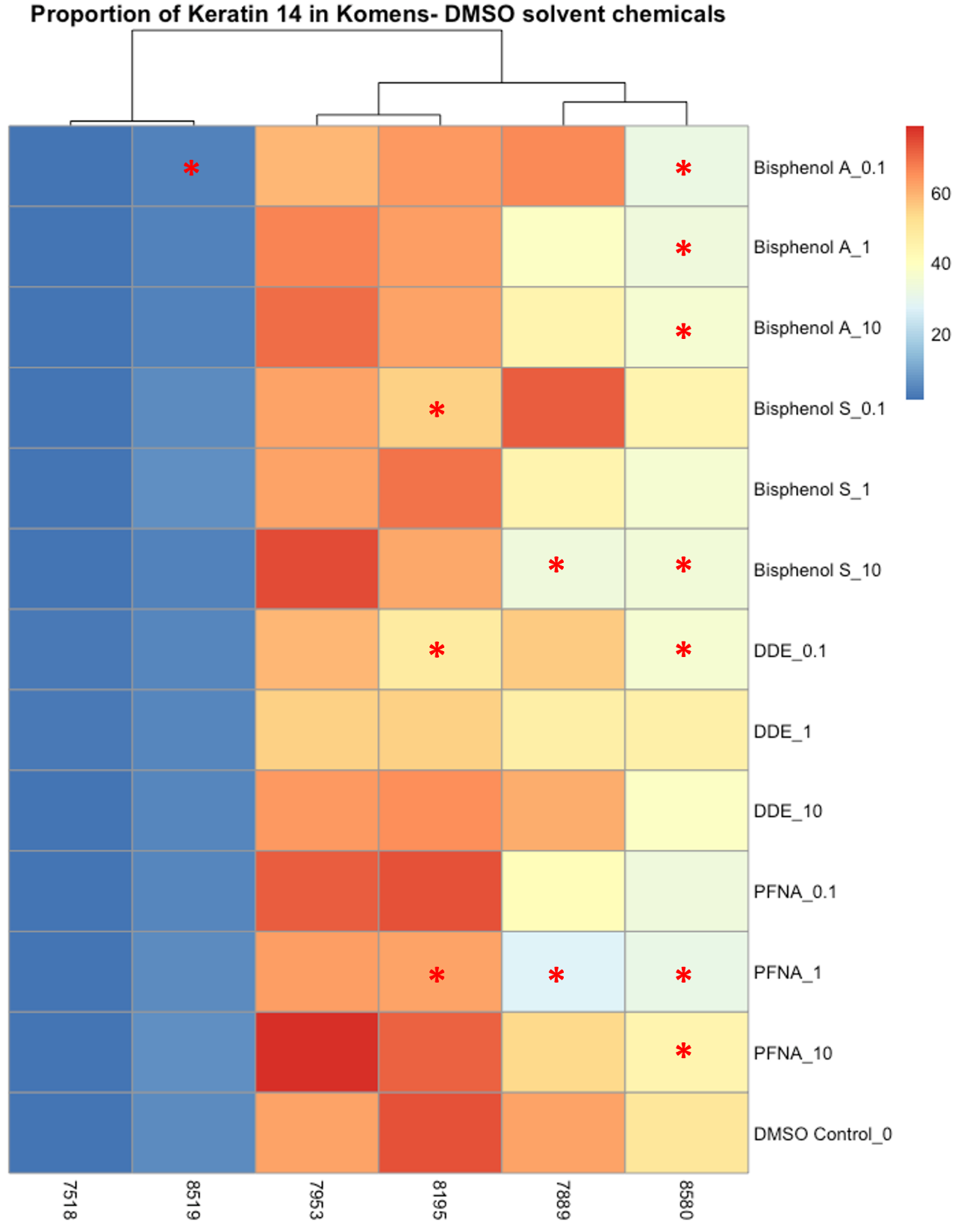
*

*Supplemental Figure 14: Heatmap depicting the percent of the cells in each treatment condition which are in a myoepithelial (KRT14 marked) state, for organic chemical treated cells only. Differences in KRT14 percentages between a given treatment and the DMSO control were determined by Wilcoxon signed rank-sum tests and denoted by a * (p<0.05). Increases in KRT14 populations are represented by a black asterisk, while decreases in KRT14 populations are represented by red asterisks.*

**
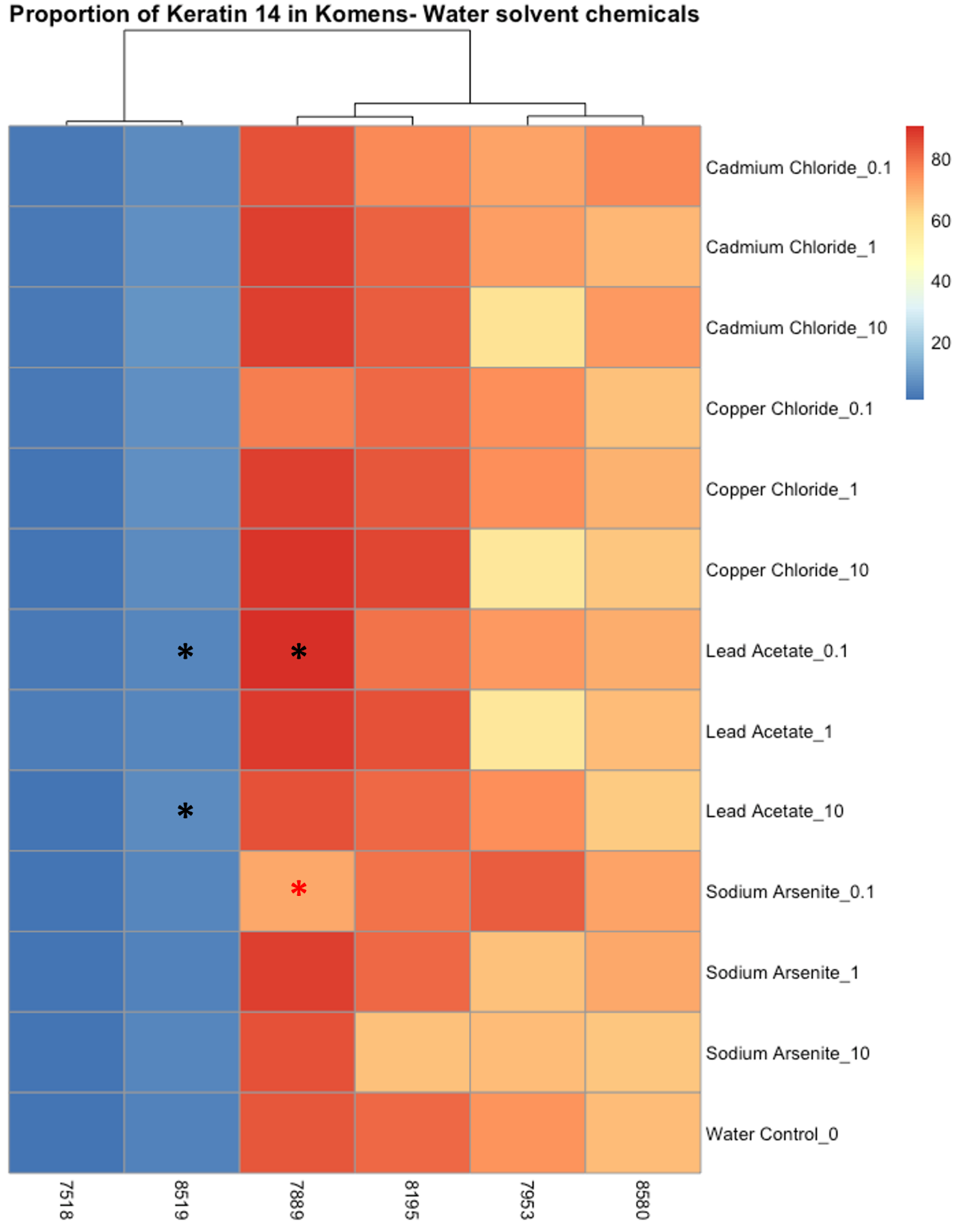
**

*Supplemental Figure 15: Heatmap depicting the percent of the cells in each treatment condition which are in a myoepithelial (KRT14 marked) state, for metal treated cells only. Differences in KRT14 percentages between a given treatment and the water control were determined by Wilcoxon signed rank-sum tests and denoted by a * (p<0.05). Increases in KRT14 populations are represented by a black asterisk, while decreases in KRT14 populations are represented by red asterisks.*


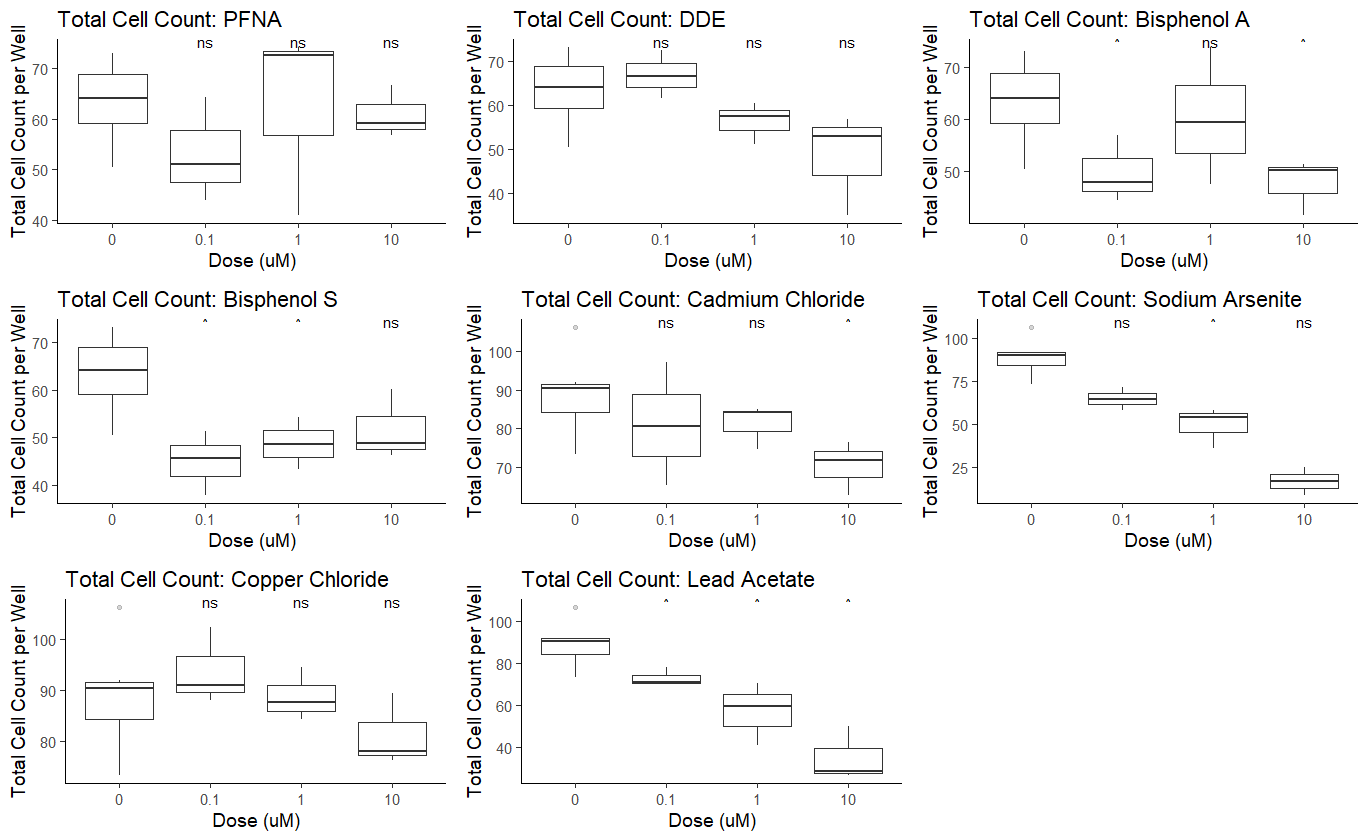


*Supplemental Figure 16: KCR 8195 total cell count for each chemical compared to each associated control. Significance determined by Wilcoxon signed rank-sum tests and denoted by a * (p<0.05).*


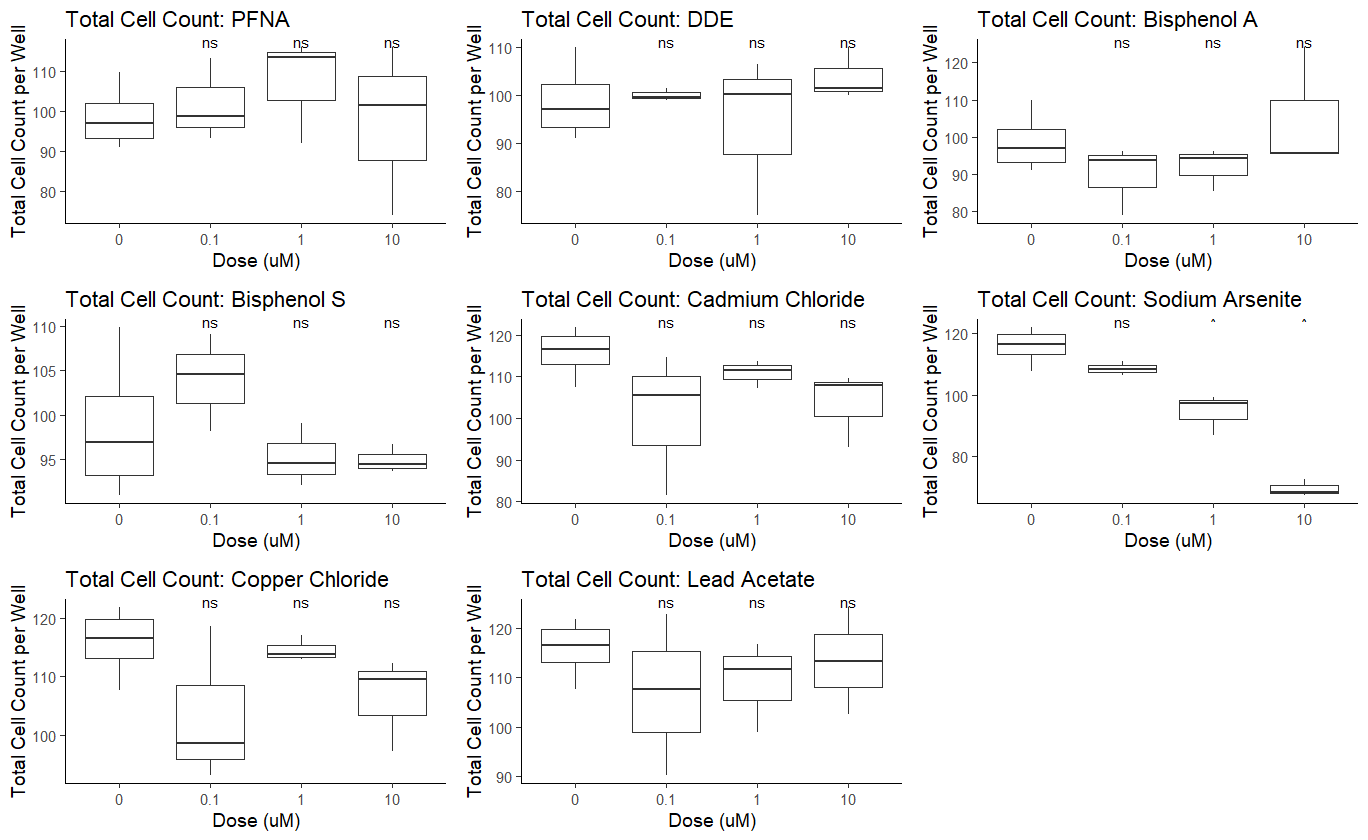


*Supplemental Figure 17: KCR 7518 total cell count for each chemical compared to each associated control. Significance determined by Wilcoxon signed rank-sum tests and denoted by a * (p<0.05).*

**
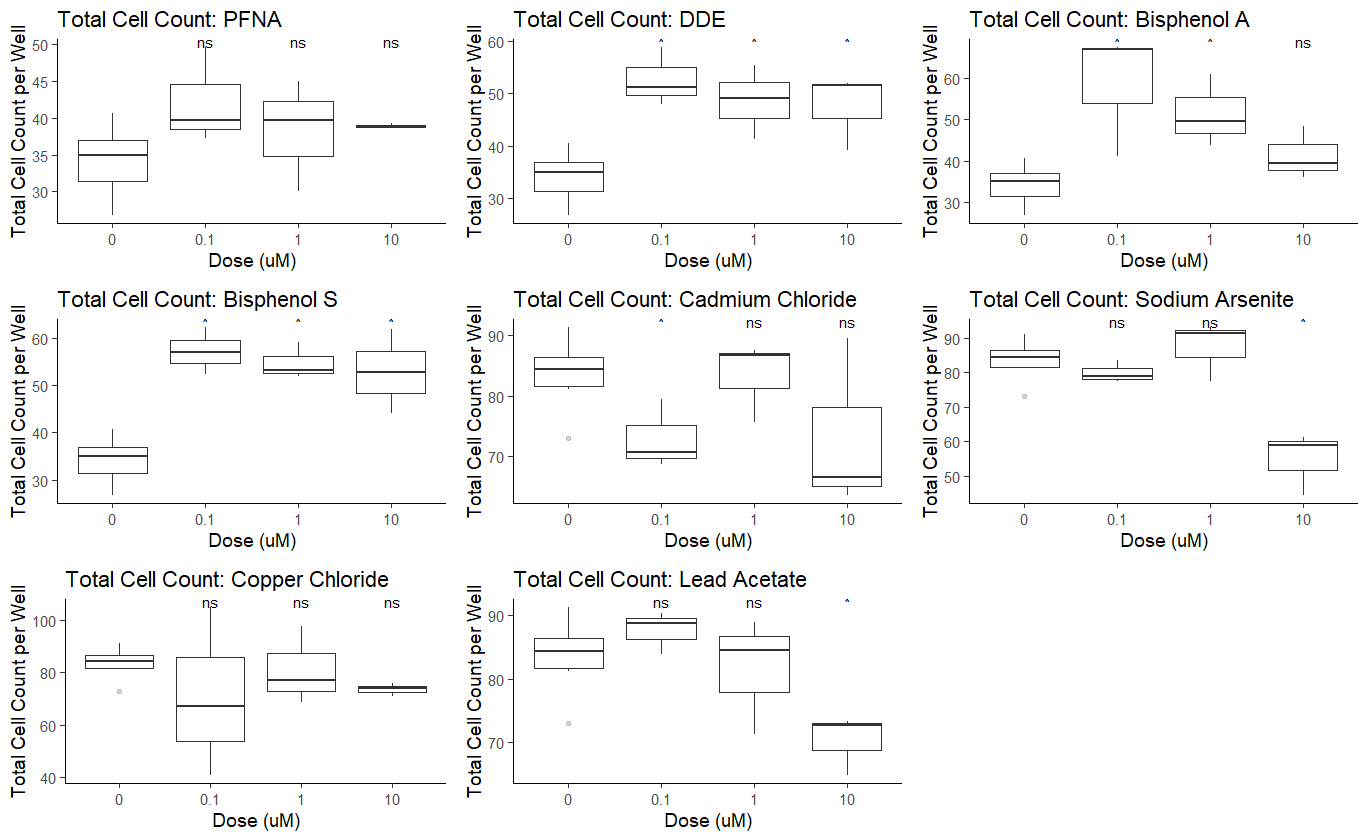
**

*Supplemental Figure 18: KCR 7889 total cell count for each chemical compared to each associated control. Significance determined by Wilcoxon signed rank-sum tests and denoted by a * (p<0.05).*


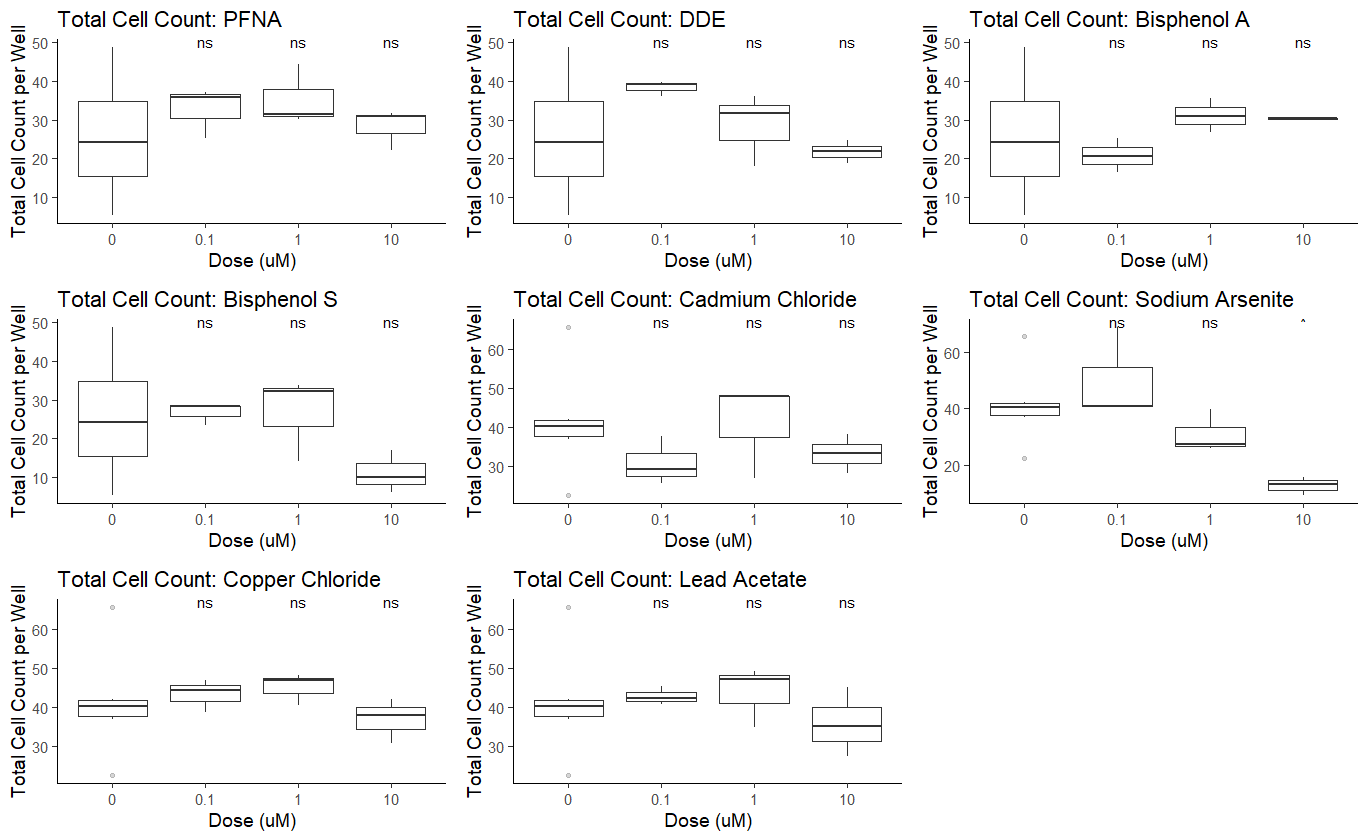


*Supplemental Figure 19: KCR 7953 total cell count for each chemical compared to each associated control. Significance determined by Wilcoxon signed rank-sum tests and denoted by a * (p<0.05).*


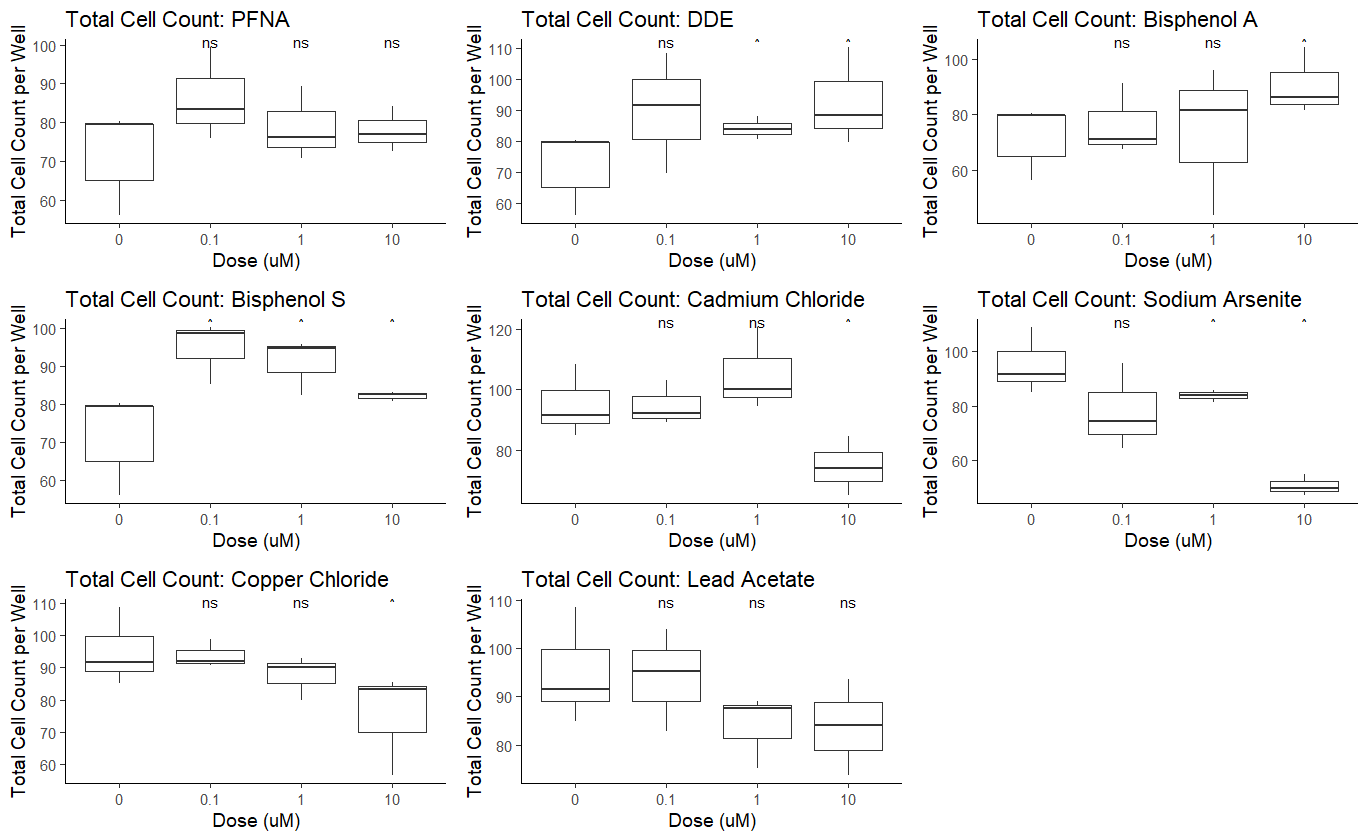


*Supplemental Figure 20: KCR 8519 total cell count for each chemical compared to each associated control. Significance determined by Wilcoxon signed rank-sum tests and denoted by a * (p<0.05).*


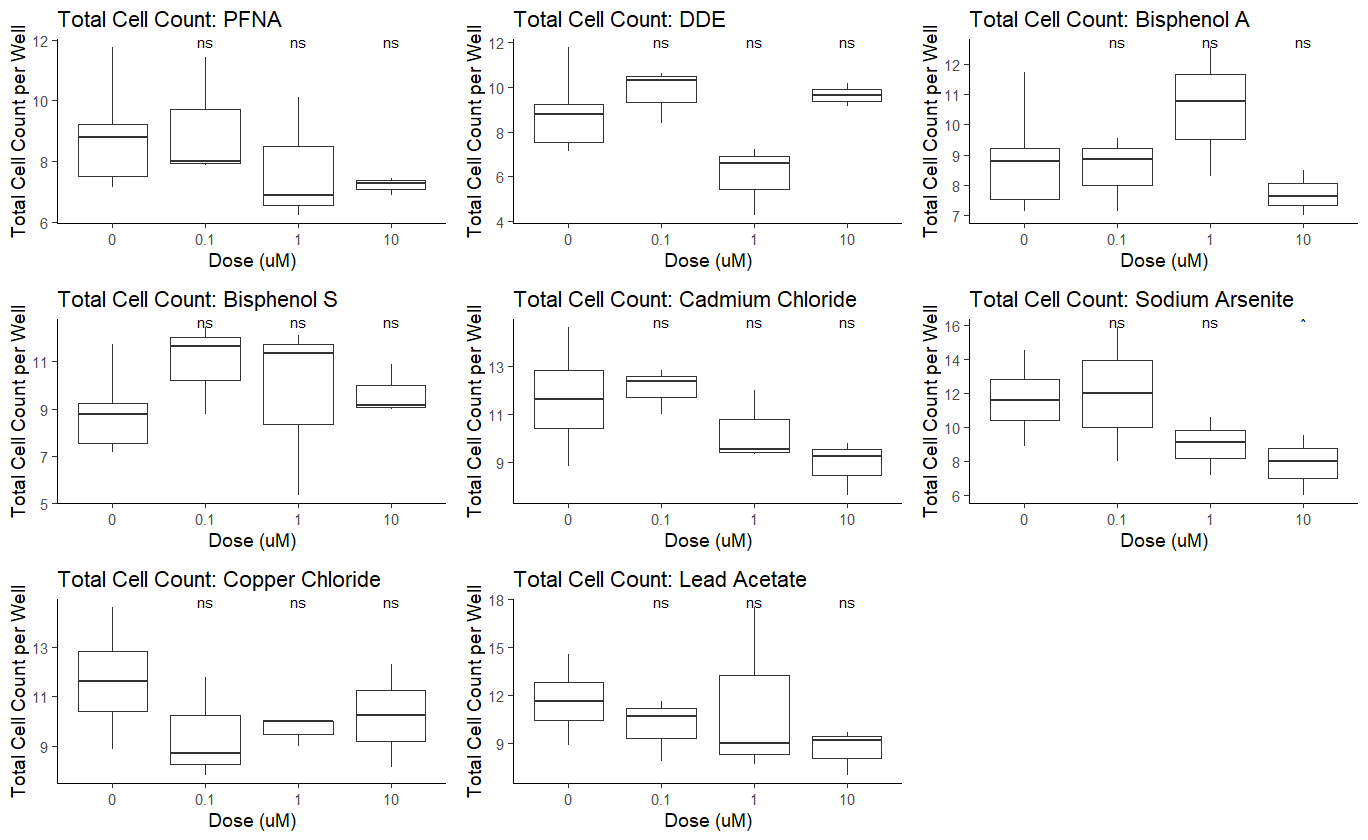


*Supplemental Figure 21: KCR 8580 total cell count for each chemical compared to each associated control. Significance determined by Wilcoxon signed rank-sum tests and denoted by a * (p<0.05).*
